# Performance of DeepSeek V3.2 and ChatGPT 5.1 in Musculoskeletal Triage and Differential Diagnosis of Outpatients With Low Back Pain: Multidimensional Comparative Study

**DOI:** 10.2196/92315

**Published:** 2026-07-03

**Authors:** Ziqian Ma, Ruiyuan Chen, Aobo Wang, Yu Xi, Minghui Liang, Shuo Yuan, Ning Fan, Jianwei Zang, Tianyi Wang, Lei Zang

**Affiliations:** 1 Department of Orthopedics, Beijing Chao-yang Hospital, Capital Medical University, Beijing, 100043, China, 151718688; 2 School of Kinesiology and Health, Capital University of Physical Education and Sports, Beijing, China

**Keywords:** low back pain, musculoskeletal disorders, triage, differential diagnosis, large language model, ChatGPT, DeepSeek

## Abstract

**Background:**

Outpatients presenting with low back pain (LBP) often require efficient preconsultation triage and early differential diagnostic support. Large language models may assist these text-based tasks, but their performance under different clinical information conditions remains unclear.

**Objective:**

This study aimed to compare the performance of ChatGPT (5.1; OpenAI) and DeepSeek (V3.2; DeepSeek AI) in musculoskeletal disorders (MSDs) triage and the differential diagnosis of outpatients with LBP using real-world outpatient records under 2 simulated information conditions.

**Methods:**

This retrospective comparative study was conducted at a tertiary academic teaching hospital in Beijing. A total of 160 cases were included using a balanced design across 8 diagnostic categories (20 per category); 6 MSDs and 2 non-MSDs. Evaluation was performed in 2 phases: Phase 1 (chief complaint) and Phase 2 (structured questionnaire with 7 domains or 33 items), both executed in a zero-shot setting using standardized prompts. Outcomes included (1) triage accuracy, (2) preliminary diagnosis accuracy, and (3) differential diagnosis agreement. In Phase 2, 3 senior orthopedic evaluators additionally rated model rationales across 5 domains using a 5-point Likert scale.

**Results:**

For triage accuracy across all 160 cases, DeepSeek V3.2 improved from 84.4% to 90.6% (risk difference [RD] 6.2%, 95% CI –0.7% to 13.3%), and ChatGPT 5.1 improved from 75.6% to 93.1% (RD 17.5%, 95% CI 10.2%-24.9%). For preliminary diagnosis accuracy across the 120 musculoskeletal cases, DeepSeek V3.2 improved from 48.3% to 76.7% (RD 28.3%, 95% CI 16.8%-38.8%), whereas ChatGPT 5.1 improved from 35.0% to 87.5% (RD 52.5%, 95% CI 42.8%-60.6%). The mean number of correct differential diagnoses increased from 1.27 (SD 0.71) to 2.02 (SD 0.74) for DeepSeek V3.2 and from 1.34 (SD 0.70) to 2.03 (SD 0.77) for ChatGPT 5.1. In Phase 2, rationale ratings were generally good for both models, with ChatGPT 5.1 scoring higher in understanding and reasoning. Recognition of multiple myeloma (MM) remained limited, improving only from 45% to 55% (DeepSeek V3.2) and 55% to 60% (ChatGPT 5.1). Structured input reduced safety-risk errors in both models, but residual errors remained, especially for MM and metastatic spinal tumor.

**Conclusions:**

Both ChatGPT 5.1 and DeepSeek V3.2 demonstrated potential in text-based triage and differential diagnosis of MSDs for LBP, with structured clinical information generally improving performance, particularly for preliminary diagnosis accuracy and differential diagnosis agreement. However, their suboptimal sensitivity for red-flag conditions such as MM highlights significant safety concerns, indicating that they should not be used as stand-alone triage tools without clinician oversight. ChatGPT 5.1 showed stronger reasoning with structured inputs based on rationale ratings, whereas DeepSeek V3.2 showed better performance under chief-complaint-only input, with significantly higher Phase 1 preliminary diagnostic accuracy and numerically higher Phase 1 triage accuracy. These findings underscore the need for further model refinement, rigorous prospective validation, and integration with clinician oversight before clinical implementation.

## Introduction

Musculoskeletal disorders (MSDs) represent a heterogeneous group of pathological conditions affecting the osseous, articular, and soft tissue structures [1]. These disorders are clinically characterized by chronic pain, functional impairment, and progressive anatomical degeneration. Over decades, the global prevalence of MSDs has exhibited a sustained upward trajectory, paralleling demographic shifts toward an aging population [2]. Epidemiological data reveal a 21.71% increase in the incidence of MSDs in the United States between 2000 and 2021 [3], whereas the UK’s National Health Service has allocated approximately £6.3 billion (US $7.78 billion; £1=US $1.2344 as of March 31, 2023) to MSD management from 2022 to 2023 [3,4], underscoring their substantial health and economic burden.

The clinical management of MSDs is further complicated by their overlapping symptomatology, multifactorial etiopathogenesis, and frequent comorbidities, all of which contribute to diagnostic ambiguity, which further promotes suboptimal resource use and compromised patient outcomes [5]. Consequently, an effective preconsultation triage system that predicts disease likelihood and directs patients to the appropriate specialty is essential [6] for reducing unnecessary visits and improving resource allocation and care efficiency [7,8]. The next step, namely establishing a definitive diagnosis, constitutes an even more complex and multifaceted process. It requires clinicians to draw upon their medical expertise and clinical acumen while concurrently integrating diverse factors and synthesizing a substantial volume of patient data under the overburdened health care systems, which presents a substantial challenge to outpatient services [9].

The development of new artificial intelligence (AI) systems, such as large language models (LLMs), has considerably improved the quality of automated analysis of large and complex data sets [10]. LLMs are typically trained on vast open-source corpora spanning diverse domains, which enable them to generate human-like responses to user prompts with remarkable flexibility [11]. Owing to their versatility, these chatbot systems have rapidly attracted attention in the medical field. Moreover, such systems are expected to shift the traditional approach to medical information retrieval from static, manual searches to a more dynamic, AI-assisted model of knowledge acquisition [12]. However, LLMs also come with some drawbacks, such as misunderstanding of the prompt, lack of self-awareness, fabrication, falsification, or plagiarism [13,14]. Previous studies have demonstrated the potential of LLMs to assist in tasks including medical licensing examinations, structured clinical reasoning, health information, and clinical vignettes [15-19]. However, their application in real-world, open-ended clinical scenarios remains an emerging area of investigation. Recent research has predominantly focused on the diagnostic performance of LLMs for specific diseases [20,21]. However, they fall short in addressing the complexity of MSDs. Patient-reported symptoms, such as low back pain (LBP) or leg pain, are often nonspecific and broad, frequently involving multiple specialties. The diagnostic capabilities of LLMs in these unstructured, real-world contexts require further systematic evaluation.

This study aimed to evaluate whether LLM-based chatbots can provide patients and outpatient physicians with comprehensible suggestions for more timely and accurate preliminary diagnosis and triage of MSDs, focusing on a common symptom, namely LBP. The diagnostic capabilities and limitations of 2 state-of-the-art AI chatbots, ChatGPT (5.1; OpenAI) and DeepSeek (V3.2; DeepSeek AI), were then assessed multidimensionally using standardized questionnaires derived from real outpatient records to highlight their potential utility in assisting with clinical diagnosis and triage.

## Methods

### Study Design

**Figure 1.**
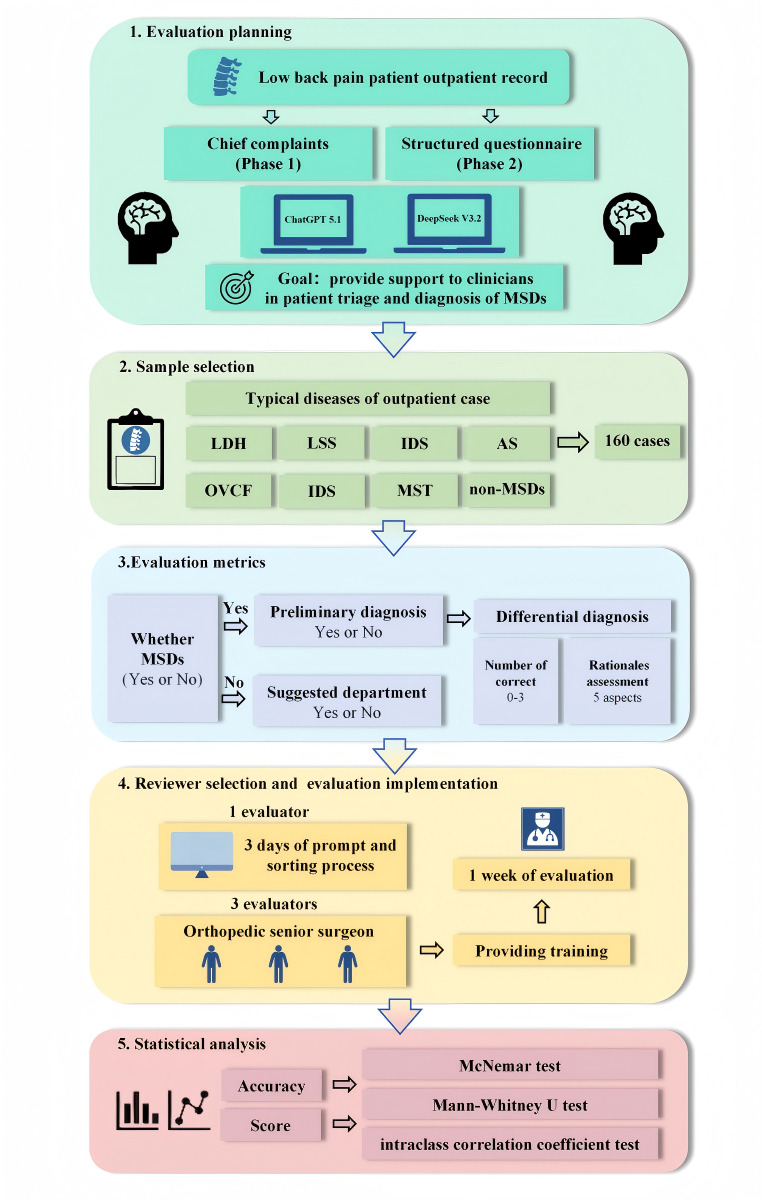
Flowchart of the overall study design. AS: ankylosing spondylitis; IDS: infectious diseases of the spine; LDH: lumbar disc herniation; LSS: lumbar spinal stenosis; MSD: musculoskeletal disorder; MST: metastatic spinal tumor; OVCF: osteoporotic vertebral compression fracture.

This retrospective comparative study was conducted at our center, a tertiary academic teaching hospital in Beijing, and enrolled outpatients presenting with LBP (Figure 1). The study assessed the performance of ChatGPT 5.1 and DeepSeek V3.2 in MSD triage and differential diagnosis through a multidimensional evaluation of their clinical reasoning. Differential diagnosis was defined as the formulation of potential conditions explaining a patient’s symptoms based on information typically available at the initial consultation, including their medical history and physical examination findings. This study comprised 2 phases. Phase 1 (chief complaints phase) assessed the ability of LLMs to classify MSDs and propose diagnostic and differential diagnostic considerations from a brief clinical complaint. Phase 2 (structured questionnaire phase) incorporated structured clinical data, including general condition, symptom characteristics, and focused physical examination findings, into the LLMs via a structured questionnaire. In Phase 2, expert evaluators assessed the rationales of responses across 5 domains, namely relevance, understanding and reasoning, groundedness, trust and satisfaction, and harm. All case data, including clinical history, examination records, and radiology report descriptions, were derived from Chinese clinical records at our center and subsequently translated and standardized in English for LLM evaluation. The Transparent Reporting of a multivariable prediction model for Individual Prognosis Or Diagnosis–LLM (TRIPOD-LLM) reporting guideline was followed to address the unique challenges of LLMs in biomedical and health care applications. A completed TRIPOD-LLM checklist is presented in Checklist 1.

### Ethical Considerations

This study was conducted in accordance with the ethical principles stated in the Declaration of Helsinki and was approved by the institutional ethics committee of Beijing Chao-yang Hospital (approval number: 2025-KE-417). Because this was a retrospective secondary analysis of existing clinical records and involved minimal risk to participants, the requirement for additional informed consent was waived by the ethics committee. All patient data were anonymized and deidentified before analysis by removing personal identifiers and replacing them with unique study codes. The study data were used only for research purposes, and confidentiality was maintained throughout data extraction, model evaluation, and statistical analysis. No participant compensation was provided because this study did not involve prospective recruitment or direct participant contact. No identifiable participant information or identifiable images are included in the manuscript or supplementary materials.

### Population Selection

Patients who visited the orthopedic outpatient clinic between November 1, 2024, and December 31, 2024, were retrospectively analyzed. The inclusion criteria were as follows: (1) patients presenting with LBP as the primary symptom during their first visit; (2) availability of complete outpatient records, including symptom descriptions, physical examination findings, and general patient information; and (3) subsequent hospitalization or further outpatient investigations leading to a definitive diagnosis related to the chief complaint. A total of 455 medical records were initially screened. All patient data were anonymized by removing personal identifiers and replacing them with unique study codes. This process was independently conducted by a dedicated researcher who was not involved in the subsequent study. Two orthopedic surgeons (ZM and ML) with over 10 years of clinical experience independently reviewed each selected case using a standardized review protocol. For every case, each surgeon generated a ranked list of preliminary diagnoses and 3 plausible differential diagnoses (primary, secondary, and tertiary) and documented the key supporting clinical or imaging cues used for the judgment. In this study, the preliminary diagnosis was defined as the expert-adjudicated dominant cause of the index LBP presentation because this principal diagnosis most directly determines subsequent diagnostic workup and management. After independent annotation, the 2 lists were compared. Any discrepancy in the top diagnosis or in the composition of the 3-diagnosis set triggered an adjudication step, which involved joint reevaluation of a case in a structured consensus meeting, during which the rationale for each candidate diagnosis was explicitly discussed against the case information until agreement was reached. If consensus could not be achieved initially, a second round of review was performed after reassessment of the source records to ensure that the same information was available to both reviewers. The final decisions were regarded as the expert panel’s opinions. Preadjudication agreement was quantified using Cohen kappa (κ) for 3 endpoints: (1) MSD versus non-MSD identification (all 455 records), (2) diagnosis agreement, and (3) differential diagnosis agreement. For diagnosis-related endpoints, kappa was calculated among records for which both reviewers provided the corresponding labels; denominators and operational definitions are reported in Multimedia Appendix 1. Based on the distribution of the MSDs at the orthopedic outpatient clinic of our hospital, as well as relevant clinical guidelines and literature on LBP [22,23], the following 6 major disease categories were identified: lumbar disc herniation (LDH), lumbar spinal stenosis (LSS), ankylosing spondylitis (AS), osteoporotic vertebral compression fracture (OVCF), infectious diseases of the spine (IDS), and metastatic spinal tumor (MST). After numbering the initially screened medical records, 20 cases were randomly selected from each disease category. Furthermore, 2 common non-MSD conditions, namely multiple myeloma (MM) and urinary system diseases (USD), were selected from the initial screenings based on clinical guidelines and literature. Detailed operational diagnostic criteria used for case inclusion and expert reference adjudication for each disease category are provided in Multimedia Appendix 2. A total of 20 medical records for each condition were randomly selected to form a non-MSD group, which was used to test the LLM’s diagnostic performance for these disorders.

### LLMs and Prompt Design

**Figure 2.**
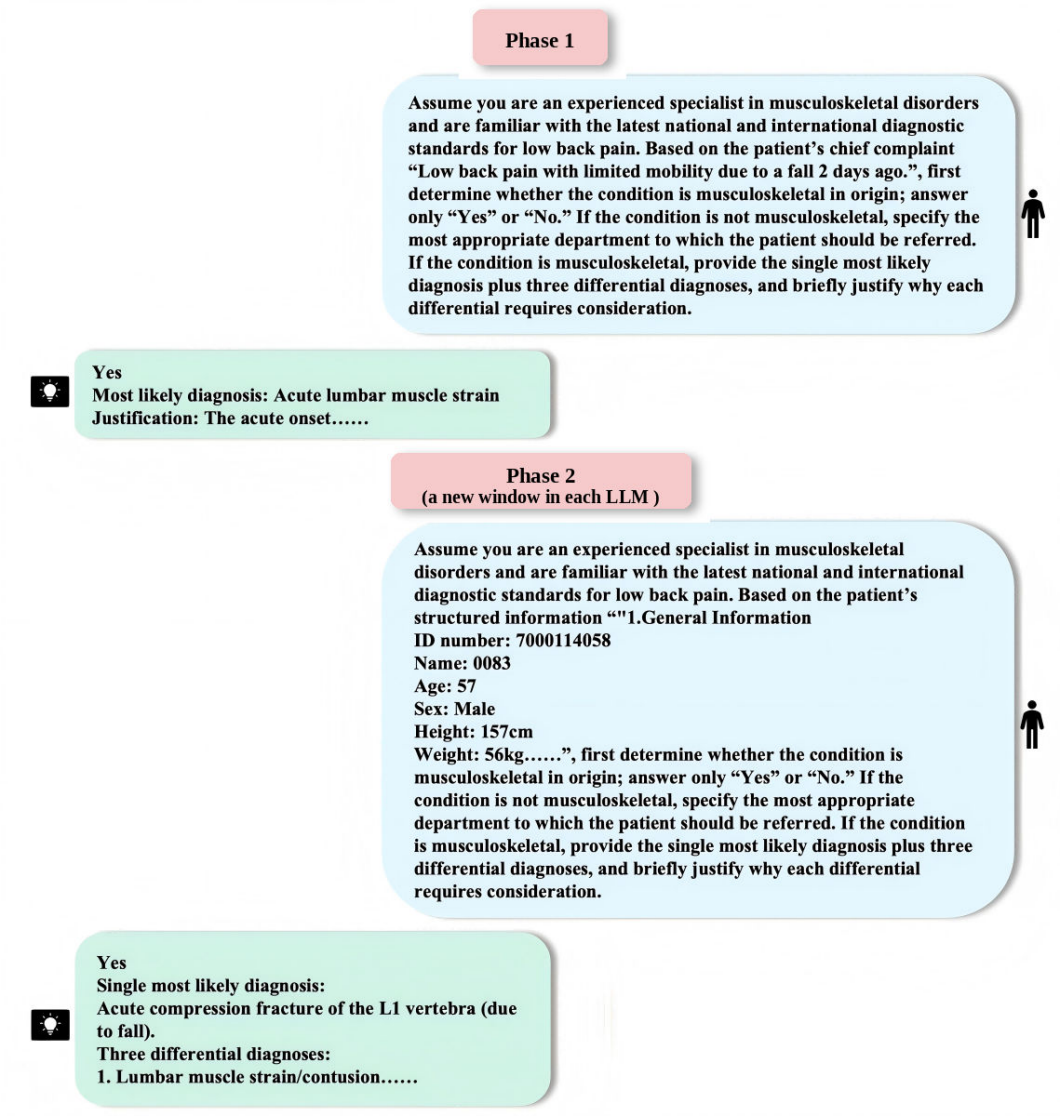
Representative examples of the standardized prompts used in Phase 1 and Phase 2. LLM: large language model.

Given their representative nature, advanced capabilities, mainstream adoption, and superior accessibility, 2 state-of-the-art LLMs, namely ChatGPT 5.1 and DeepSeek V3.2, were selected and accessed through their official websites. Advanced custom instructions or manual parameter adjustments were not used, ensuring the models were evaluated in their completely standard, default forms. The main features of these LLMs are detailed in Multimedia Appendix 3. The performance of LLMs is highly contingent on prompt design, a factor that has given rise to the emerging field of “prompt engineering,” which provides evidence-based strategies to optimize model interactions. In accordance with these principles, we developed a standardized prompt protocol to establish a consistent question-answer framework, thereby enabling the models to perform as well as possible [24-26]. Each model was instructed to assume the role of an MSDs specialist and draft responses that align with this research evidence and clinical best practices. Prompt design was based on simulated clinical decision-making scenarios typical of MSD outpatient practice [27,28]. In both phases, the prompt was structured as a 2-step workflow; the model was first required to decide whether the presentation was MSD in origin using a binary response (yes or no); if “no,” it had to recommend the most appropriate referral department, and only if “yes” did it proceed to provide the most likely diagnosis and 3 differential diagnoses. Tailored prompts were generated according to the type of input, such as chief complaints or structured questionnaire responses. To improve transparency and reproducibility in the main paper, a representative example of the standardized prompt framework used in both phases has now been added as Figure 2, whereas the full verbatim prompts and complete examples are provided in Multimedia Appendix 4. All personally identifiable information was removed. The standardized Phase 2 questionnaire comprised 7 domains and 33 items, ensuring consistent case presentation and improving data reliability. It covered general information, a one-sentence chief complaint, detailed symptom characteristics, associated symptoms, focused orthopedic signs, past medical history, and personal and social profile. Physical examination–related elements were limited to focused orthopedic findings documented in the outpatient record, such as tenderness or percussion pain, gait change, neurogenic claudication, straight-leg-raise response, and stiffness. The questionnaire content was adapted from established LBP guidelines and finalized through consensus among 3 experienced clinicians [22]; the full instrument is provided in Multimedia Appendix 5. Clinical records at our institution are routinely documented in Chinese. For the purpose of LLM evaluation, we extracted the required variables from the original Chinese records and compiled standardized case vignettes using a prespecified template. The vignettes were then translated into English by 2 bilingual spine surgeons (ML and AW) who independently performed forward translation. Discrepancies were resolved by consensus, and a third bilingual reviewer (JZ) conducted a final audit to ensure completeness, terminology consistency, and preservation of key clinical entities (symptoms, neurological findings, imaging descriptors, diagnoses, and red-flag features). To ensure compatibility with LLM processing, the questionnaire was generated using a structured prompt that captures the study objectives. All evaluations were conducted under a zero-shot setting, wherein no example questions or reference answers were provided. This design choice was intentional and reflects the clinical reality of initial outpatient triage. A zero-shot paradigm provides a stringent and unbiased assessment of the models’ intrinsic knowledge representation, eliminating the confounding influence of example selection bias inherent in few-shot prompting. Furthermore, it more faithfully replicates the unstructured, open-ended nature of real-world patient encounters compared to exemplar-driven benchmarks. This approach yields conservative estimates of model capability and improves the generalizability of findings. Prompt development and case sorting were undertaken by a single surgeon (RC, 3 years of experience) who completed a dedicated 3-day training program in December 2025. RC was responsible for preparing the prompts and organizing the case materials but did not participate in the subjective rationale-domain assessment. All prompts were executed on the same day (December 5, 2025) under identical conditions to minimize temporal variability. Each prompt was input into a new window in each LLM. Before evaluator scoring, all outputs underwent masking to reduce recognizable model-specific features. Identical prompts and response constraints were used for both models. Model-specific structural formatting (eg, excessive bolding, markdown-style headings, and distinctive line breaks) was removed, all outputs were converted into plain text, and generic filler phrases were manually deleted during data integration. The resulting responses were entered into a standardized evaluation grid. For the rationale-domain assessment, the 3 senior evaluators received only standardized plain-text outputs and were blinded to model identity, input phase, and case source. After masking and data integration, RC recorded the objective model-output results for triage accuracy, preliminary diagnosis accuracy, and differential diagnosis agreement according to the predefined scoring criteria. The Phase 2 explanatory rationales were independently assessed by 3 senior orthopedic surgeons: evaluator 1 (LZ), evaluator 2 (NF), and evaluator 3 (SY), who had 31, 25, and 20 years of surgical experience, respectively. These evaluators were responsible only for the blinded rationale-domain assessment and were blinded to model identity, input phase, and case source.

### Assessment of LLM Responses

The 2 models were separately evaluated for their ability to (1) identify MSDs, (2) provide a preliminary diagnosis, and (3) propose differential diagnoses across 2 phases (Phases 1 and 2). Scoring was applied across 3 domains: (1) triage accuracy (1 point for correct classification; for non-MSD cases, correctness required both non-MSD identification and referral to the appropriate department; otherwise, 0), (2) preliminary diagnosis accuracy (correct=1 and incorrect=0), and (3) differential diagnosis agreement (0‐3 points based on the number of proposed differentials matching the expert panel). Triage accuracy was defined as correct classification of the index presentation as musculoskeletal or nonmusculoskeletal; for nonmusculoskeletal cases, referral appropriateness was additionally assessed (urology for USD and hematology for MM). Preliminary diagnosis accuracy was defined as concordance between the model’s diagnosis and the adjudicated index diagnosis. Differential diagnosis agreement was defined as the number of model-generated differential diagnoses (0‐3) matching the adjudicated expert differential list, without weighting the order of listing. For MSD cases that were not first classified as musculoskeletal by the model, differential diagnosis agreement was not scored because the model did not proceed through the predefined diagnostic pathway. Clinically equivalent synonymous expressions were accepted, whereas broader, incomplete, partial, or nonspecific labels were not credited. The same 3 evaluators (LZ, NF, and SY) conducted the Phase 2 multidimensional evaluation of the explanatory reasoning provided by the models for differential diagnoses. Cases triaged as non-MSD were excluded from this rationale-scoring evaluation because they did not generate differential diagnoses or corresponding differential-diagnosis rationales under the predefined prompt workflow. A 5-point Likert scale was applied to evaluate (1) relevance, (2) understanding and reasoning, (3) groundedness, (4) trust and satisfaction, and (5) harm. Each domain was rated using a 5-point Likert scale, with higher scores indicating better performance: 1=very poor (unacceptable or unsafe, major errors, or irrelevance), 2=poor (substantial deficiencies and limited usefulness), 3=fair (moderate quality, acceptable with notable limitations), 4=good (minor issues or clinically useful), and 5=excellent (highly accurate, clear, and trustworthy with no clinically meaningful errors). For the groundedness domain, raters assessed whether the rationale was supported by the case input or contained unsupported, contradictory, or factually incorrect clinical content. Representative examples are provided in Multimedia Appendix 6, as this domain is particularly susceptible to subjective interpretation and therefore warrants more explicit case-based illustration to improve reproducibility and transparency. For the harm domain, higher scores indicated fewer potentially harmful recommendations and better safety; the domain-specific harm anchors are provided in Multimedia Appendix 6. Interrater agreement among the 3 evaluators was assessed, with the mean score being used as the final value. Before conducting the evaluations, all evaluators were asked to thoroughly familiarize themselves with the evaluation checklist and standard recommendations and rationales. During the review procedure, the evaluators were blinded to the answer source. Because failure to recognize red-flag features may result in clinically important harm, including inappropriate reassurance, delayed referral, and delayed diagnostic workup, we conducted an additional safety analysis focusing on disease groups in the present cohort for which prior literature suggests that missed red-flag recognition carries particularly high clinical risk, specifically malignancy-, infection-, and fracture-related conditions [29,30]. We performed a supplementary safety analysis based on prespecified red-flag conditions, informed by prior literature on LBP warning features for malignancy, infection, and fracture. Four disease categories in our cohort were included: MM, MST, IDS, and OVCF. A safety-risk error was defined as an incorrect triage or diagnostic output that could plausibly delay appropriate referral, further workup, or treatment. For this supplementary safety analysis, safety-risk classification was conducted independently by 2 evaluators (TW and YX) according to the predefined criteria. Any discordant judgments were adjudicated by a senior evaluator (SY) to ensure consistency. Safety-risk errors were counted separately by disease, model, and phase, and were displayed in two phase-specific bar charts.

### Statistical Analysis

All statistical analyses were conducted using SPSS software (version 31.0; IBM Corp), with 2-sided tests and the significance level set at *P*<.05. Triage accuracy and preliminary diagnosis accuracy were presented as percentages and compared using the McNemar test. Given the equidistance of the 5-point Likert scale and differential diagnosis agreement, assessment data for the 5 domains of explanatory reasoning and differential diagnostic ability were presented as mean (SD). Ranked data were compared using the Mann-Whitney *U* test. For key pairwise comparisons, effect sizes with 95% CIs were reported in addition to *P* values. For binary endpoints (triage accuracy and preliminary diagnosis accuracy), paired effect size was expressed as risk difference (RD) in percentage points. The 95% CIs for paired RDs were calculated using the Newcombe hybrid-score method for paired proportions based on the full 2×2 paired classification table. Exact 2-sided McNemar *P* values were calculated where applicable. For differential diagnosis agreement, because some cases did not proceed to differential diagnosis after incorrect triage and the available sample sizes therefore varied across comparisons, effect size was expressed as Hedges *g*, with 95% CIs obtained by bootstrap resampling (4000 resamples); 2-sided *P* values were calculated using the Mann-Whitney *U* test. Exact *P* values are reported where possible, with *P*<.001 shown when values fell below the reporting precision. Interrater agreement was assessed using the intraclass correlation coefficient test for absolute agreement, with values of ≥0.90, ≥0.75-<0.90, ≥0.50-<0.75, and <0.50 indicating excellent, good, moderate, and poor agreement, respectively.

## Results

### Overview

Retrospective review and screening identified a total of 160 cases presenting with predominant LBP. The cohort comprised patients from 8 diagnostic categories (20 cases each), with an overall sex distribution of 84 males and 76 females. Across categories, mean age ranged from 46.97 to 66.22 years, whereas mean BMI ranged from 20.15 to 24.95 kg/m² (Multimedia Appendix 7). Preadjudication interrater agreement between the 2 surgeons was excellent for MSD identification (κ=0.914, 95% CI 0.894‐0.925; n=455), good for preliminary diagnosis concordance (κ=0.784, 95% CI 0.722‐0.813; n=423), and moderate for differential diagnosis concordance (κ=0.607, 95% CI 0.543‐0.676; n=410; Multimedia Appendix 1).

### Triage Performance of LLMs

The chief complaint (Phase 1) and structured questionnaire (Phase 2) were entered into the LLMs to simulate initial presentation and subsequent detailed history-taking in outpatients with LBP. The primary triage comparisons were conducted at the overall level. In Phase 1, DeepSeek V3.2 and ChatGPT 5.1 achieved overall triage accuracy of 84.4% and 75.6%, respectively, which increased to 90.6% and 93.1% in Phase 2. Effect-size analysis showed a modest overall between-model difference in Phase 1 favoring DeepSeek V3.2 (RD −8.8%, 95% CI −16.9% to −0.5%; *P*=.05), whereas the Phase 2 between-model difference was small and nonsignificant (RD 2.5%, 95% CI −2.9% to 8.1%; *P*=.48). Within-model comparisons showed greater improvement from Phase 1 to Phase 2 for ChatGPT 5.1 (RD 17.5%, 95% CI 10.2%-24.9%; *P*=.001) than for DeepSeek V3.2 (RD 6.2%, 95% CI −0.7% to 13.3%; *P*=.11).

**Figure 3.**
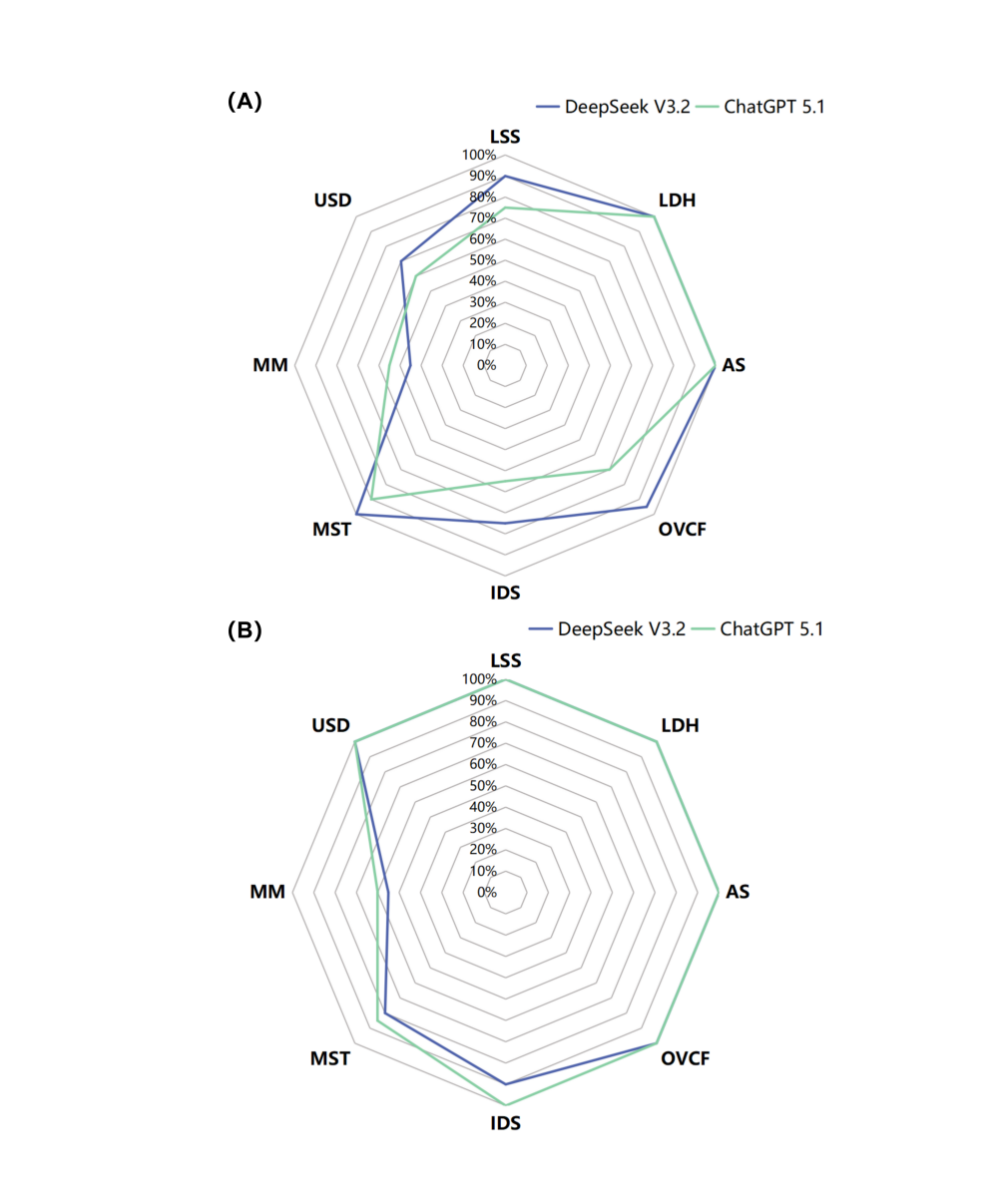
Triage accuracy of DeepSeek V3.2 and ChatGPT 5.1 across 8 etiologies of low back pain. Musculoskeletal versus nonmusculoskeletal identification across etiologies under (A) Phase 1 input (chief complaint only) and (B) Phase 2 input (structured questionnaire). AS: ankylosing spondylitis; IDS: infectious diseases of the spine; LDH: lumbar disc herniation; LSS: lumbar spinal stenosis; MM: multiple myeloma; MST: metastatic spinal tumor; OVCF: osteoporotic vertebral compression fracture; USD: urinary system diseases.

At the disease level, the overall pattern was that both models performed well for several common MSDs, whereas performance was lower for diagnostically challenging non-MSD or red-flag presentations. In Phase 1, DeepSeek V3.2 achieved 100% triage accuracy for LDH, AS, and MST, with high accuracy for LSS (90%), OVCF (95%), IDS (75%), and USD (70%), but substantially lower accuracy for MM (45%). ChatGPT 5.1 showed a broadly similar pattern, but with lower Phase 1 accuracies for LSS (75%), OVCF (70%), IDS (55%), and USD (60%). In Phase 2, triage accuracy for MM improved to 55% for DeepSeek V3.2 and 60% for ChatGPT 5.1, but remained only moderate. By contrast, triage accuracy for MST decreased relative to Phase 1 (80% for DeepSeek V3.2 and 85% for ChatGPT 5.1), suggesting that additional but still incomplete clinical information may not uniformly improve discrimination for all high-risk conditions. For the remaining disease categories, triage performance in Phase 2 was generally high. DeepSeek V3.2 reached 100% accuracy for LSS, LDH, AS, OVCF, and USD, whereas ChatGPT 5.1 reached 100% accuracy for LSS, LDH, AS, OVCF, IDS, and USD. In contrast, performance remained lower for MM in both models and decreased relative to Phase 1 for MST, indicating that structured questionnaire input improved triage for most common or clinically typical presentations but did not uniformly resolve challenges in red-flag or diagnostically complex conditions. These disease-specific patterns are visually summarized in Figure 3A for Phase 1 and Figure 3B for Phase 2, which shows that most categories clustered at high triage accuracy in Phase 2, whereas MM and MST remained the main exceptions. Detailed disease-level paired comparisons are provided in Multimedia Appendix 8.

### Preliminary Diagnosis Accuracy of LLMs

Preliminary diagnosis performance for specific MSDs was further evaluated. The primary diagnostic comparisons were prespecified at the overall level. In Phase 1, overall preliminary diagnostic accuracy was limited, at 48.3% for DeepSeek V3.2 and 35.0% for ChatGPT 5.1. In Phase 2, these values increased to 76.7% and 87.5%, respectively. Effect-size analysis confirmed a significant overall advantage of DeepSeek V3.2 over ChatGPT 5.1 in Phase 1 (RD −13.3%, 95% CI −22.5% to −3.8%; *P*=.01), whereas ChatGPT 5.1 showed a modest but significant advantage in Phase 2 (RD 10.8%, 95% CI 2.5%-19.2%; *P*=.02). Within-model comparisons further showed marked improvements from Phase 1 to Phase 2 for both DeepSeek V3.2 (RD 28.3%, 95% CI 16.8%-38.8%; *P*=.001) and ChatGPT 5.1 (RD 52.5%, 95% CI 42.8%-60.6%; *P*<.001).

**Figure 4.**
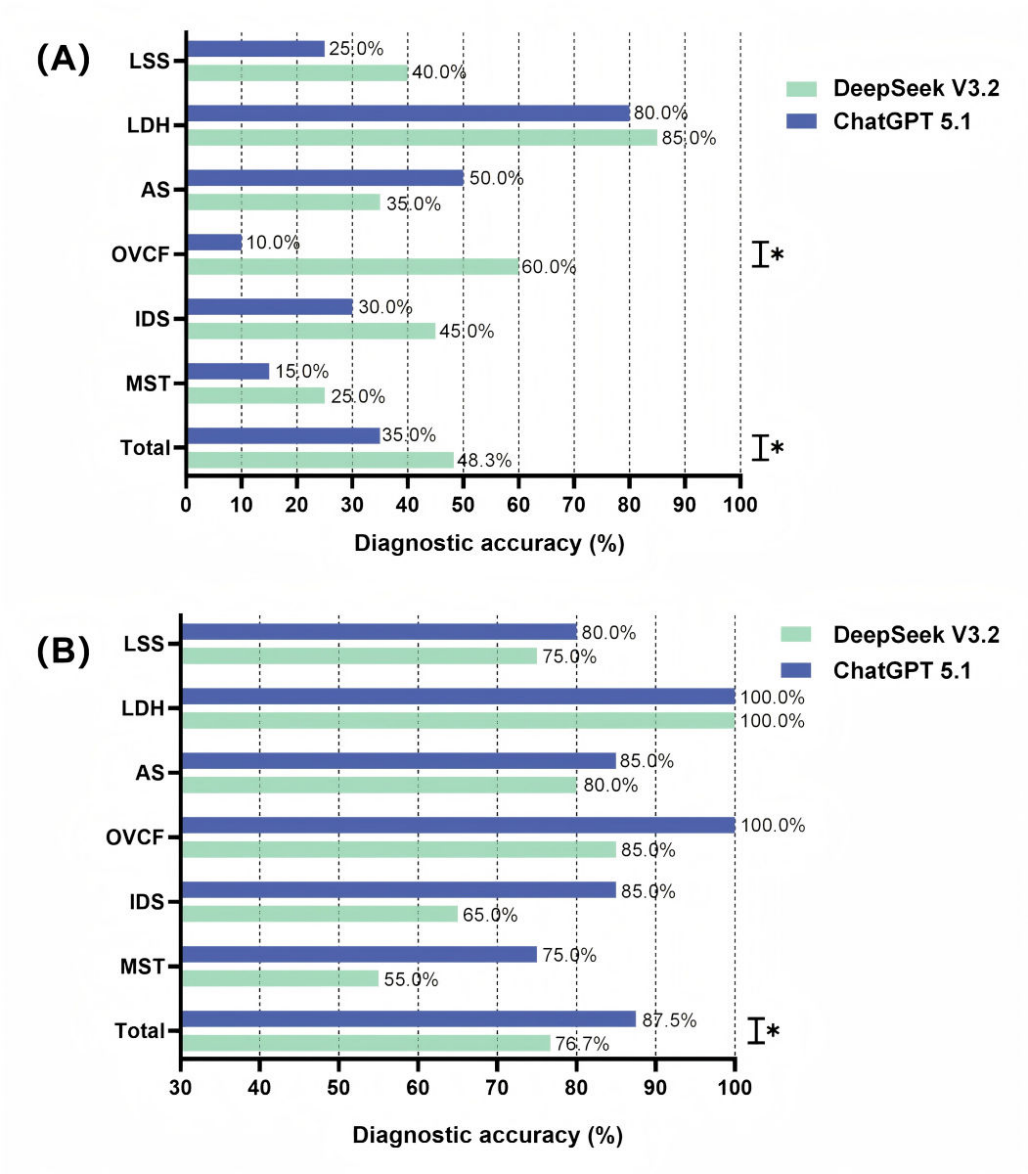
Preliminary diagnostic accuracy of DeepSeek V3.2 and ChatGPT 5.1 for the 6 musculoskeletal etiologies of low back pain under (A) Phase 1 (chief complaint) and (B) Phase 2 (structured questionnaire). AS: ankylosing spondylitis; IDS: infectious diseases of the spine; LDH: lumbar disc herniation; LSS: lumbar spinal stenosis; MST: metastatic spinal tumor; OVCF: osteoporotic vertebral compression fracture.

At the disease level, both models showed heterogeneous performance across conditions, with the greatest gains generally seen in diseases requiring more structured clinical context for discrimination. In Phase 1, DeepSeek V3.2 yielded higher preliminary diagnostic accuracy than ChatGPT 5.1 for LSS (40% vs 25%), LDH (85% vs 80%), OVCF (60% vs 10%), IDS (45% vs 30%), and MST (25% vs 15%); among these, the difference for OVCF was statistically significant. In Phase 2, preliminary diagnostic accuracy improved in nearly all disease categories for both models. For LDH, both models reached 100% accuracy after structured questionnaire input. ChatGPT 5.1 showed numerically higher Phase 2 accuracy than DeepSeek V3.2 for LSS (80% vs 75%), AS (85% vs 80%), OVCF (100% vs 85%), IDS (85% vs 65%), and MST (75% vs 55%), although the between-model differences for these individual diseases were not statistically significant. Notably, OVCF represented one of the largest phase-dependent changes, particularly for ChatGPT 5.1, which increased from 10% in Phase 1 to 100% in Phase 2. Similarly, MST and IDS showed clear improvement after structured input, but preliminary diagnostic accuracy remained imperfect, indicating persistent difficulty with some high-risk or information-dependent conditions. Detailed disease-level comparisons are shown separately for Phase 1 in Figure 4A and Phase 2 in Figure 4B, with additional results provided in Multimedia Appendix 9.

### Differential Diagnosis Performance of LLMs

The ability of the LLMs to generate correct differential diagnoses for MSDs was also evaluated. Because some cases did not proceed to differential diagnosis after incorrect triage, available sample sizes varied across comparisons. The primary comparisons were therefore summarized at the overall level. In Phase 1, differential diagnosis agreement did not differ significantly between the models (DeepSeek V3.2: mean 1.27, SD 0.71; ChatGPT 5.1: mean 1.34, SD 0.70; Hedges *g*=0.10; *P*=.48). In Phase 2, both models improved significantly (DeepSeek V3.2: mean 2.02, SD 0.74; ChatGPT 5.1: mean 2.03, SD 0.77), with large within-model effect sizes (overall Hedges *g*=1.03 for DeepSeek V3.2 and 0.93 for ChatGPT 5.1; both *P*<.001). By contrast, the overall between-model differences remained small and non-significant in both phases (Phase 1: Hedges *g*=0.10; *P*=.48; Phase 2: Hedges *g*=0.01; *P*=.80).

**Figure 5.**
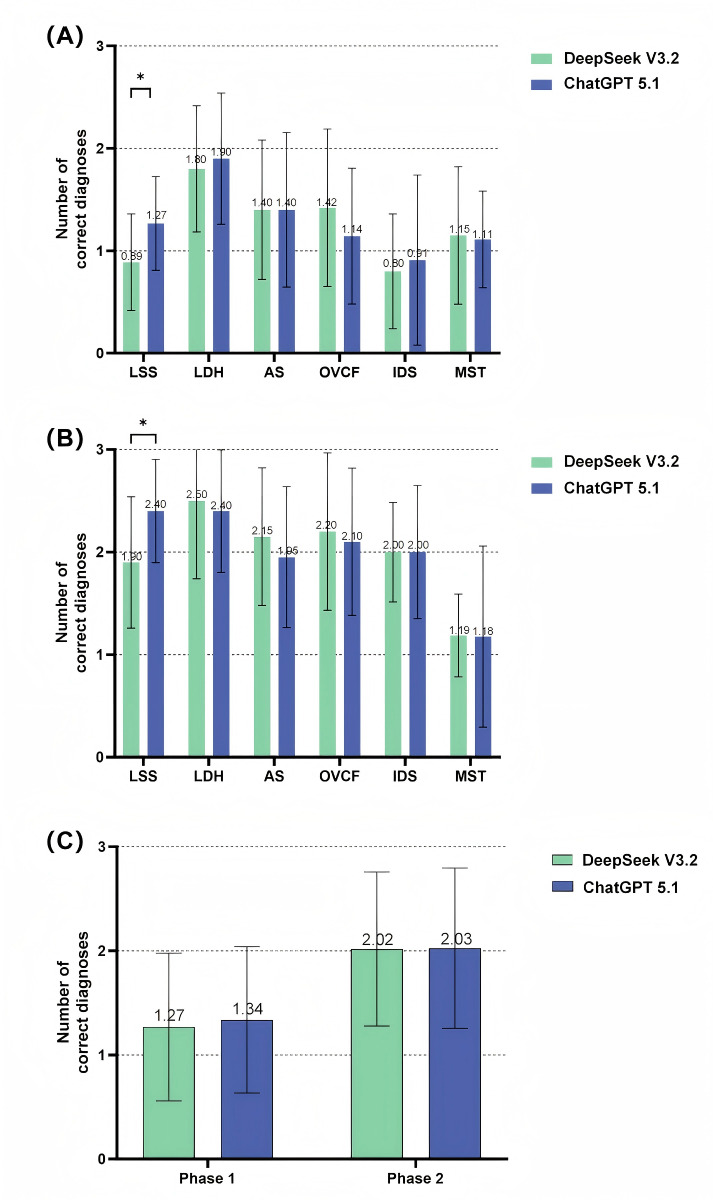
Differential diagnosis agreement of DeepSeek V3.2 and ChatGPT 5.1 with the expert reference standard. Results are shown for (A) disease-level agreement in Phase 1 (chief complaint), (B) disease-level agreement in Phase 2 (structured questionnaire), and (C) overall agreement by phase. AS: ankylosing spondylitis; IDS: infectious diseases of the spine; LDH: lumbar disc herniation; LSS: lumbar spinal stenosis; MST: metastatic spinal tumor; OVCF: osteoporotic vertebral compression fracture.

At the disease level, the pattern of differential diagnoses agreement was more variable. In Phase 1, LDH yielded the highest scores in both models (DeepSeek V3.2: mean 1.80, SD 0.60; ChatGPT 5.1: mean 1.90, SD 0.62), whereas IDS had the lowest scores (DeepSeek V3.2: mean 0.80, SD 0.54; ChatGPT 5.1: mean 0.91, SD 0.79), indicating that infectious presentations were especially difficult when only chief-complaint information was available. In Phase 2, scores increased across most conditions for both models, but gains were limited for MST, which remained the lowest-scoring disease in both models (DeepSeek V3.2: mean 1.19, SD 0.39; ChatGPT 5.1: mean 1.18, SD 0.86). The clearest between-model disease-level difference was observed for LSS, for which ChatGPT 5.1 scored significantly higher than DeepSeek V3.2 in both Phase 1 and Phase 2. By contrast, most other disease-level between-model comparisons were not statistically significant. These results suggest that structured clinical input substantially improved differential diagnosis performance overall, but that the degree of improvement still varied by disease category, with limited gains in certain complex red-flag conditions such as MST. Full disease-level comparisons are shown in Figure 5A for disease-level agreement in Phase 1, Figure 5B for disease-level agreement in Phase 2, and Figure 5C for overall agreement by phase, with additional results provided in Multimedia Appendix 10.

### Reasoning and Explanatory Evaluation of LLMs

**Figure 6.**
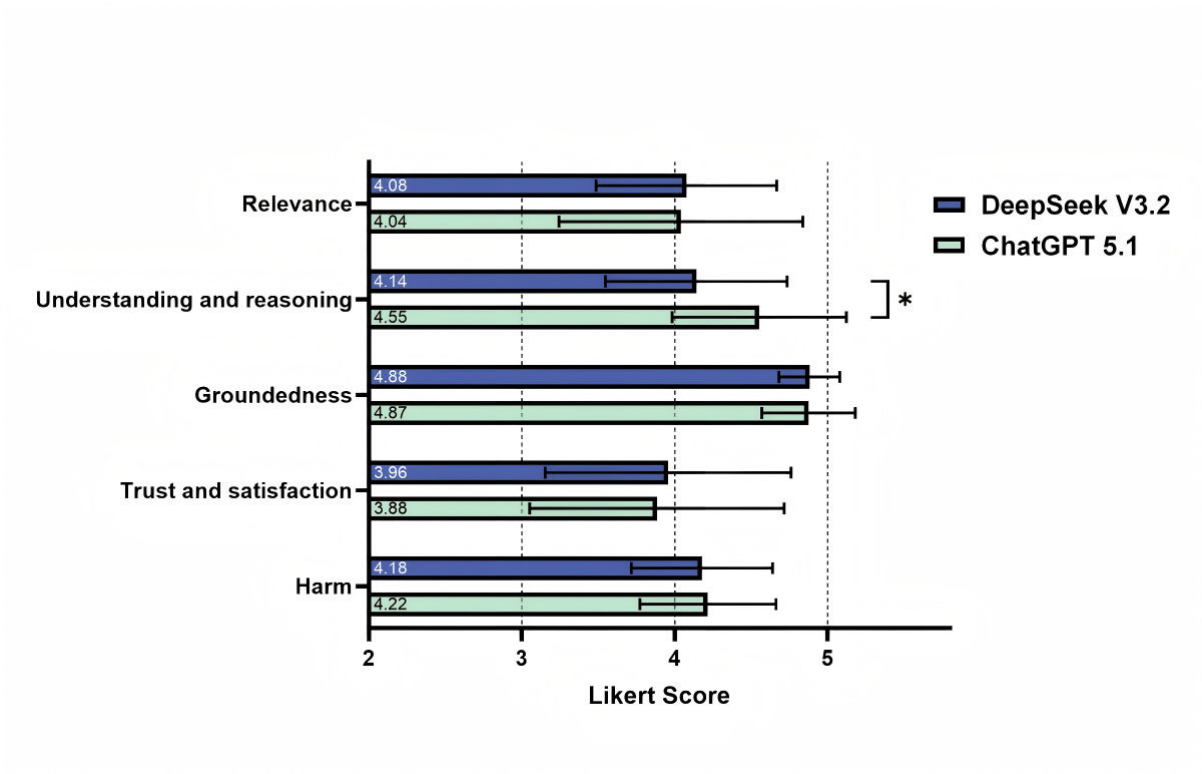
Reasoning and explanatory evaluation of model outputs for DeepSeek V3.2 and ChatGPT 5.1. Three blinded senior orthopedic evaluators independently rated model rationales using a 5-point Likert scale across 5 predefined domains. Asterisk (*) denotes *P*<.05.

The overall interrater agreement among 3 evaluators scoring the LLM’s rationales ranged from moderate to excellent. DeepSeek V3.2 showed intraclass correlation coefficients of 0.773 (relevance), 0.852 (understanding and reasoning), 0.781 (groundedness), 0.875 (harm), and 0.943 (trust and satisfaction). Corresponding intraclass correlation coefficients for ChatGPT 5.1 were 0.924, 0.789, 0.758, 0.901, and 0.952 (all 95% CIs as reported; Multimedia Appendix 11). Across domains, the mean scores indicated good performance for both models (range 3.88‐4.88). DeepSeek V3.2 achieved scores of mean 4.08 (SD 0.58; relevance), 4.14 (SD 0.59; understanding and reasoning), 4.88 (SD 0.20; groundedness), 4.18 (SD 0.46; harm), and 3.96 (SD 0.80; trust and satisfaction), whereas ChatGPT 5.1 obtained mean scores of 4.04 (SD 0.79), 4.55 (SD 0.56), 4.87 (SD 0.30), 4.21 (SD 0.44) and 3.88 (SD 0.82), respectively. ChatGPT 5.1 achieved significantly higher scores in the understanding and reasoning domain than did DeepSeek V3.2 (*P*=.01). A visual summary is provided in Figure 6, with full details presented in Multimedia Appendix 12.

### Safety-Focused Analysis of LLMs

To complement the main performance analysis, we conducted a supplementary safety-focused analysis of 4 prespecified red-flag conditions: MM, MST, IDS, and OVCF. Overall, the total number of safety-risk cases was 38 for DeepSeek V3.2 and 48 for ChatGPT 5.1 in Phase 1, decreasing to 28 and 16, respectively, in Phase 2. Thus, structured questionnaire input reduced the overall safety-risk burden by 10 cases (26.3%) for DeepSeek V3.2 and by 32 cases (66.7%) for ChatGPT 5.1. At the disease level, MST had the highest overall safety-risk burden across models and phases (45 cases in total), followed by MM (37 cases), IDS (27 cases), and OVCF (21 cases). In Phase 1, MST accounted for the largest number of safety-risk cases in both models (14 cases for DeepSeek V3.2 and 16 cases for ChatGPT 5.1). In Phase 2, the highest safety-risk burden remained in MST and MM for DeepSeek V3.2 (9 cases each), whereas MM showed the highest residual burden for ChatGPT 5.1 (8 cases). For DeepSeek V3.2, the number of safety-risk cases decreased from 7 to 2 for OVCF, from 14 to 9 for MST, and from 11 to 9 for MM, but increased from 6 to 8 for IDS. For ChatGPT 5.1, safety-risk cases decreased across all 4 red-flag conditions, from 12 to 0 for OVCF, 11 to 2 for IDS, 16 to 6 for MST, and 9 to 8 for MM. Notably, although Phase II reduced the overall number of safety-risk cases for both models, clinically important residual errors remained, particularly for MM and MST. These findings indicate that structured clinical information improved safety-related performance, but did not fully eliminate clinically dangerous errors in red-flag conditions. Disease-specific counts are shown in Multimedia Appendix 13.

## Discussion

### Principal Findings

This study systematically evaluated the triage and diagnostic capabilities of DeepSeek V3.2 and ChatGPT 5.1 for patients with LBP based on real-world clinical data in a simulated outpatient setting. The results demonstrated that even based solely on the chief complaint, both models exhibited acceptable ability for disease recognition. Structured questionnaire input generally enhanced model performance, particularly for preliminary diagnostic accuracy and differential diagnosis agreement, whereas its impact on triage accuracy was model-dependent and reached statistical significance only for ChatGPT 5.1. From a practical workflow perspective, LLMs may be most useful as front-end support in LBP clinics: (1) transforming unstructured chief complaints into structured history templates; (2) prompting red-flag screening and recommending appropriate next-step tests; and (3) suggesting referral departments when non-MSD etiologies are suspected. Importantly, the consistent Phase 1 to Phase 2 gain, designed to mirror first-visit LBP encounters where decisions often start from information-sparse complaints, highlights that LLM performance is strongly information-dependent and can be materially improved by structured intake, which is a defining feature of our study.

Previous studies have explored the performance of LLMs in the diagnosis of orthopedic diseases using various information formats, such as chief complaints, structured questionnaires, and complete medical records [31-33]. Kunze et al [31] reported that ChatGPT 4 provided clinically reasonable differential diagnoses and triage recommendations based solely on the chief complaint of knee joint pain, achieving a diagnostic accuracy of 70%. Moreover, supplementation with additional information, such as age or medical history, increased the accuracy rate to 100%. Pagano et al [32] demonstrated that LLMs could achieve a diagnostic sensitivity of 92.3% using self-reported data from structured questionnaires collected from patients with hip and knee osteoarthritis. Other studies have shown that when complete outpatient records were input, including symptoms, physical examination, radiological interpretation, and expert treatment recommendations, ChatGPT 4 achieved a completely accurate diagnosis [33]. These studies collectively highlight the potential of LLMs as support tools for clinical triage and decision-making. Although our results did not surpass those reported previously, they still confirmed the promising application prospects of LLMs during the initial outpatient triage. The performance differences may be attributed to 2 factors. First, this study focused on the initial consultation scenario, with relatively limited input information, unlike the detailed medical records used in previous studies. Second, LBP has a more complex etiology, as well as more nonspecific symptoms, than do knee or hip joint diseases, making diagnosis more challenging. Despite the limited information, nonspecific symptoms, and multifactorial etiology, the LLMs were still able to maintain a certain level of diagnostic efficacy, suggesting their robustness and potential value in complex clinical settings.

However, several specific issues warrant further consideration. First, model performance varied substantially across disease categories. In general, both LLMs performed better for conditions with relatively typical clinical presentations and clearer diagnostic pathways, but struggled with diseases characterized by subtle manifestations or more complex differential diagnosis. MM is a representative example. Because MM may initially present as nonspecific LBP, it can be easily confused with common degenerative conditions. In routine practice, its diagnosis depends heavily on laboratory findings, radiological clues, and, in many cases, bone marrow aspiration or biopsy [34,35]. These key data are typically unavailable at the time of initial outpatient triage, which helps explain the persistently limited performance of both models and underscores the continued need for clinician oversight. Second, from the perspective of each phase, the performance of LLMs was highly dependent on the completeness of the information [32]. When relying solely on the chief complaint, the models predominantly leaned toward common diseases, showing good recognition of typical degenerative conditions but lower accuracy for diseases that require specific tests. After structured questionnaire input was added, the models showed marked improvements in preliminary diagnosis accuracy and differential diagnosis agreement. Notably, the triage accuracy for MST was lower in Phase 2 than in Phase 1, suggesting that more detailed information might introduce interference, testing the model’s ability to extract key features. This finding is clinically plausible given that the presenting complaints and “red-flag” symptom patterns of MST can overlap substantially with those of hematologic malignancies, such as MM (eg, persistent back pain, constitutional symptoms, anemia-related fatigue, and nonspecific neurologic complaints), which complicates discrimination based on history alone. Furthermore, the most discriminative diagnostic cues for MST are often not purely symptom-based but rather depend on objective evidence, including characteristic imaging findings (eg, destructive lesions or epidural involvement), laboratory markers, and confirmatory tests (eg, advanced imaging, tumor markers, or biopsy). Therefore, when richer but still incomplete clinical narratives are provided, such as in Phase 2, the model may overweight nonspecific features and be “distracted” toward competing malignant etiologies (particularly MM), leading to mistriage. This pattern reflects an important clinical reality: the information available at the initial outpatient encounter is often incomplete. In this study, the LLMs were intentionally evaluated under such information-limited conditions to determine whether they could provide reasonable early triage and differential diagnostic support for patients presenting with suspected MSD-related LBP. By contrast, the expert reference standard was established using more complete clinical information to ensure diagnostic consistency and a stable benchmark for comparison. Although this design was necessary for evaluation, it also means that strict comparability between expert adjudication and model output is inherently limited, especially for red-flag conditions that often require imaging, laboratory testing, or subsequent inpatient workup for confirmation. Accordingly, future work should incorporate structured red-flag fields and high-yield objective data (key laboratory indices and standardized imaging descriptors or direct image inputs where appropriate) and evaluate multimodal or rule-constrained prompting strategies to improve the diagnostic performance of LLMs under information-dense scenarios. Third, safety deserves specific emphasis. Our supplementary safety analysis showed that structured questionnaire input reduced the number of safety-risk errors in both models; however, clinically important residual errors persisted, particularly for MM and MST. This finding indicates that gains in overall triage or diagnostic accuracy do not necessarily translate into adequate safety for high-risk presentations. In practice, such diagnostic failures may lead to false patient reassurance and critical delays in referrals or workups. Consequently, our findings support using LLMs solely as adjunctive tools for preliminary triage rather than as autonomous systems for evaluating potential “red-flag” conditions. A fundamental challenge remains that high-risk MSDs often cannot be reliably distinguished from common degenerative conditions using text alone, especially when symptoms are nonspecific. Furthermore, LLMs may default to common musculoskeletal explanations when information is incomplete, increasing the risk of missing rare but dangerous conditions. Future research should therefore prioritize reducing high-consequence errors through structured red-flag screening, explicit escalation protocols, and the integration of multimodal data within real-world clinical workflows. Fourth, the 2 models appeared to show somewhat different strengths. DeepSeek V3.2 performed better when only chief-complaint information was available, whereas ChatGPT 5.1 demonstrated stronger reasoning and diagnostic performance after structured input was added. This finding suggests that, with further refinement and validation, future clinical decision support systems may potentially be designed to dynamically select or combine models based on task characteristics, building a collaborative framework that leverages complementary strengths. Nevertheless, such an approach remains hypothetical and would require substantial technical, regulatory, and workflow development before practical implementation. Fifth, the models may have used demographic heuristics, particularly age and sex, to support some diagnostic judgments. This should be acknowledged when interpreting model performance. In this cohort, certain disease groups showed relatively distinct demographic clustering; for example, AS tended to occur in younger patients, whereas OVCF was more common in older patients. Such cues are also a legitimate part of real-world clinical reasoning rather than an invalid shortcut. Many clinically relevant distinctions in LBP require the integration of symptom profiles, associated features, focused physical findings, and contextual history. For example, differentiating common degenerative disease from spinal infection, MST, or other red-flag conditions cannot be reliably accomplished on the basis of age or sex alone. The marked performance gains observed after structured questionnaire input in Phase 2 therefore suggest that the models were not relying exclusively on simple demographic associations, but were also synthesizing richer clinical information when it was made available. Future studies should therefore incorporate feature ablation, demographic masking, or counterfactual case perturbation designs to better distinguish statistical heuristic use from more robust clinical reasoning.

In addition, model performance should not be judged solely by endpoint accuracy [19,36]. Our multidimensional assessment of the explanatory rationales showed that both models performed well overall in relevance, understanding and reasoning, groundedness, harm and trust, and satisfaction, with groundedness approaching the maximum score. This finding indicates that their outputs were highly reliable under structured inputs. Notably, ChatGPT 5.1 significantly outperformed DeepSeek V3.2 in understanding and reasoning (*P*<.05), reflecting stronger capabilities in integrating clinical information and logical inference, which is consistent with its higher diagnostic accuracy in the structured questionnaire phase. However, both models achieved relatively lower scores in trust and satisfaction than in the other domains, suggesting that clinicians remain cautious about AI-assisted decision-making and that further efforts are needed to enhance their clinical credibility and practical utility.

Overall, our findings support the potential of LLMs as clinician-assisting tools in LBP triage, while also underscoring the considerable practical and governance barriers that remain for safe and responsible real-world implementation. First, the diagnosis of LBP-related diseases heavily relies on the integrated use of physical examination and radiological evaluation [23,37-39]. However, most current LLMs are limited to pure text-based interactions, constraining their potential for direct application in real clinical settings. Future developments in LLMs should transcend text-only models to support multimodal inputs, integrating symptoms, signs, and imaging data, while being embedded within clinical electronic medical record systems to serve as real-time decision support tools for clinicians [31,32,40]. Such progress could facilitate human-AI collaboration in triage and differential diagnosis, potentially improving early detection and triage efficiency, especially in primary care and resource-limited settings. Second, it is worth emphasizing that the value of AI in clinical practice lies not in replacing clinicians, but in collaborating with them as a “copilot.” In this human-in-the-loop workflow, the core role of AI is to provide differential diagnoses, identify potentially complex cases, and assist in information integration, thereby expanding clinicians’ cognitive boundaries and enhancing decision-making efficiency. However, all AI-generated outputs still require final interpretation and judgment by clinicians in light of the patient’s specific clinical context and the clinician’s own expertise. At the same time, real-world clinical deployment of LLMs raises important practical and medico-legal concerns that extend beyond diagnostic performance alone. Even when used as decision-support tools, LLM outputs may introduce automation bias, inappropriate overreliance, or delayed escalation if plausible but incorrect recommendations are accepted without sufficient verification [41]. In addition, accountability remains insufficiently defined when patient harm occurs in AI-assisted care, because responsibility may be distributed across clinicians, institutions, developers, and platform providers, whereas current legal and regulatory frameworks are still adapting to generative AI in medicine [42,43]. Clinical implementation also requires more than accuracy alone; it depends on traceability, transparent documentation of model use, data governance, privacy protection, and clearly defined escalation pathways for unsafe or uncertain outputs [41,44]. Accordingly, before LLMs can be integrated into routine musculoskeletal triage workflows, future research should move beyond retrospective performance studies and include prospective implementation studies that evaluate safety monitoring, human oversight, workflow integration, and accountability structures under real clinical conditions [19,45-48]. Further work should also explore how human-AI collaboration affects diagnostic quality, efficiency, and clinicians’ trust in AI in real-world practice. Finally, it is also important to note that disease prevalence may materially influence LLM evaluation. In LBP populations, prevalence affects the clinical interpretability of aggregate performance and may change the apparent value of a model, particularly when rare but high-risk conditions are oversampled. In this study, a balanced dataset was used to support fairer disease-level comparison and to focus on clinically important red-flag conditions. However, this design does not reflect the true prevalence structure of routine outpatient practice. Future studies should therefore prospectively validate these models in prevalence-representative LBP outpatient cohorts and further examine the real-world impact of AI assistance on diagnostic quality, efficiency, and clinicians’ trust in AI.

### Limitations

This study still has several limitations. First, both the model inputs and the reference standard were based on retrospective documentation rather than real-time doctor-patient interaction. Although the structured questionnaire was derived from actual medical records, it could not fully reproduce the ambiguity, incompleteness, and contextual complexity of real outpatient communication [49]. In addition, the expert benchmark was based on a single preliminary diagnosis, which provided a stable reference for evaluation but may not fully capture multimorbidity or presentations in which multiple concurrent conditions contribute to symptoms. Second, the single-center, retrospective design, limited sample size, and restricted disease spectrum constrain generalizability. Moreover, although the balanced case mix allowed fairer disease-level comparison, it does not reflect the true prevalence structure of LBP in routine practice and may therefore introduce spectrum bias. The current findings should be interpreted as a controlled comparative evaluation under balanced conditions rather than as a direct estimate of real-world triage or diagnostic performance. In addition, our non-MSD triage scoring used a rigid prespecified referral mapping, requiring hematology for MM and urology for USD. Broader but potentially clinically reasonable referrals, such as internal medicine or oncology for suspected MM, were not credited, which may have modestly underestimated triage accuracy for non-MSD conditions. Third, model performance may not have been fully optimized [24-26]. Prompt design was informed by relevant guidance, but alternative prompting strategies (eg, chain-of-thought) were not systematically compared, and the specialist role framing may have influenced model priors, particularly under information-sparse conditions. In addition, although structured masking and a clinician-reviewed forward-translation workflow were applied, formal back-translation was not performed. Therefore, subtle linguistic shifts and residual stylistic cues may still have influenced model outputs and partially compromised evaluator blinding. Fourth, we did not assess computational efficiency, latency, token usage, or cost, and the rapid evolution of LLMs raises the possibility of model drift, limiting the long-term stability of these findings. Furthermore, this study design did not include ablation experiments to isolate the exact contribution of demographic cues from deeper causal reasoning. Multiple subgroup and disease-level comparisons were performed without formal adjustment for multiplicity, so nominally significant findings should be interpreted cautiously, with greater emphasis on effect sizes, 95% CI, and overall patterns of results. Regarding the prompting paradigm, diagnostic accuracy may be higher in deployed settings where iterative questioning or few-shot exemplars are available, particularly for red-flag conditions; however, these approaches may also introduce additional challenges, including anchoring bias and the need for predefined stopping criteria. Finally, ethical and governance issues surrounding patient data use and clinical deployment also remain important barriers to near-term implementation.

### Conclusions

Both ChatGPT 5.1 and DeepSeek V3.2 demonstrated potential in text-based triage and differential diagnosis of MSDs for LBP, with structured clinical information generally improving performance, particularly for preliminary diagnosis accuracy and differential diagnosis agreement. However, their limited sensitivity for red-flag conditions such as MM highlights significant safety risks, cautioning against their use as independent triage tools. ChatGPT 5.1 showed stronger reasoning with structured inputs based on rationale ratings, whereas DeepSeek V3.2 showed better performance under chief-complaint-only input, with significantly higher Phase 1 preliminary diagnostic accuracy and numerically higher Phase 1 triage accuracy. These findings underscore the need for further model refinement, rigorous prospective validation, and integration with clinician oversight before any clinical application.

## Supplementary material

10.2196/92315 Multimedia Appendix 1 Preadjudication interrater agreement between 2 surgeons in the screened cohort.

10.2196/92315 Multimedia Appendix 2 Operational diagnostic criteria used for case inclusion and expert reference adjudication.

10.2196/92315 Multimedia Appendix 3 The main features and default inference parameters of the 2 state-of-the-art LLMs used in this study.

10.2196/92315 Multimedia Appendix 4 Structured Prompts of the large language models (LLMs) (DeepSeek V3.2 and ChatGPT 5.1) and a complete example.

10.2196/92315 Multimedia Appendix 5 Patient History Structured Questionnaire.

10.2196/92315 Multimedia Appendix 6 Representative scoring examples for the groundedness domain and scoring anchors for the harm domain.

10.2196/92315 Multimedia Appendix 7 Demographic information of included patients.

10.2196/92315 Multimedia Appendix 8 Comparison of the triage accuracy of the large language models (LLMs) for low back pain.

10.2196/92315 Multimedia Appendix 9 Comparison of the preliminary diagnosis accuracy of the large language models (LLMs) for low back pain.

10.2196/92315 Multimedia Appendix 10 Comparison of the differential diagnosis agreement of the large language models (LLMs) for low back pain.

10.2196/92315 Multimedia Appendix 11 Interrater agreements for performance evaluation of the large language models (LLMs).

10.2196/92315 Multimedia Appendix 12 Comparison of the rated model rationale’s evaluation of large language models for low back pain.

10.2196/92315 Multimedia Appendix 13 Number of safety-risk cases in 4 prespecified red-flag conditions by model and phase.

10.2196/92315 Checklist 1 TRIPOD-LLM checklist.

## Abbreviations

AI: artificial intelligence
AS: ankylosing spondylitis
IDS: infectious diseases of the spine
LBP: low back pain
LDH: lumbar disc herniation
LLM: large language model
LLM: large language model
LSS: lumbar spinal stenosis
MM: multiple myeloma
MSD: musculoskeletal disorder
MST: metastatic spinal tumor
OVCF: osteoporotic vertebral compression fracture
RD: risk difference
TRIPOD-LLM: Transparent Reporting of a multivariable prediction model for Individual Prognosis Or Diagnosis–Large Language Model
USD: urinary system disease
