# Supplementary material for: Performance of DeepSeek V3.2 and ChatGPT 5.1 in Musculoskeletal Triage and Differential Diagnosis of Outpatients With Low Back Pain: Multidimensional Comparative Study
Source: J Med Internet Res. 2026 Jul 3;28:e92315. doi: 10.2196/92315 (PMC13331072; doi:10.2196/92315)
Supplement: Multimedia Appendix 1 [file jmir-v28-e92315-s001.docx]

**Multimedia Appendix 2.** Pre-adjudication inter-rater agreement between two surgeons in the screened cohort.

| Agreement endpoint | κ (95% CI) ^a^ | Denominator(n) ^b^ |
| --- | --- | --- |
| Triage accuracy | 0.914(0.894-0.925) | 455 |
| Preliminary diagnosis accuracy | 0.784(0.722-0.813) | 423 |
| Differential diagnosis agreement | 0.607(0.543-0.676) | 410 |

^a^ 95% confidence intervals (CIs) for Cohen’s κ were obtained using nonparametric bootstrap resampling at the record level (B = 2000 resamples). For each resample, n records were drawn with replacement and κ was recomputed; the 2.5th and 97.5th percentiles of the bootstrap distribution were used as the 95% CI.

^b^ Denominator denotes the number of screened records for which both surgeons provided a preliminary diagnosis and a top-3 differential diagnosis list, respectively.

Operational definitions:

2 surgeons independently reviewed all 455 screened records prior to adjudication. Cohen’s kappa (κ) was computed for triage accuracy directly. For preliminary diagnosis and differential diagnoses, each surgeon’s assessment was coded as binary concordance with the expert panel’s opinions, and κ was calculated between surgeons based on these indicators. Records lacking the corresponding labels were excluded from the relevant κ calculation.
