# Supplementary material for: Performance of DeepSeek V3.2 and ChatGPT 5.1 in Musculoskeletal Triage and Differential Diagnosis of Outpatients With Low Back Pain: Multidimensional Comparative Study
Source: J Med Internet Res. 2026 Jul 3;28:e92315. doi: 10.2196/92315 (PMC13331072; doi:10.2196/92315)
Supplement: Multimedia Appendix 2 [file jmir-v28-e92315-s002.docx]

**Multimedia Appendix 3**. Operational diagnostic criteria used for case inclusion and expert reference adjudication

The reference standard in this study was an adjudicated clinical reference diagnosis based on retrospective review of the complete available records related to the index presentation. To improve transparency and consistency, the following operational criteria were used during case selection and expert adjudication. These criteria were intended to support stable disease classification within the study and should be interpreted as study-specific operational definitions rather than uniform gold-standard criteria applied identically across all disease categories. In particular, only cases with diagnoses already clearly established through prior clinical care and subsequent diagnostic evaluation were eligible for inclusion. For each case, adjudication was based on the most reliable diagnosis supported by the available outpatient documentation and, where applicable, subsequent inpatient records, imaging findings, laboratory results, specialist assessments, and final diagnostic conclusions from further workup. When available and clinically relevant, operative records and documented intraoperative findings were also reviewed and used to clarify the final diagnosis.

1. Lumbar disc herniation (LDH)

Cases were classified as LDH when the available records supported a clinical syndrome of low back pain and/or radicular lower-extremity symptoms consistent with lumbar nerve root irritation, together with imaging findings compatible with disc herniation at a clinically corresponding level. Supporting evidence included 1 or more of the following: dermatomal leg pain, sensory disturbance, focal motor weakness, or nerve tension signs documented in the record, plus MRI or CT evidence of disc protrusion, extrusion, or sequestration judged relevant to the index presentation.

2. Lumbar spinal stenosis (LSS)

Cases were classified as LSS when the records supported a clinical syndrome compatible with degenerative lumbar spinal stenosis, typically including neurogenic claudication and/or posture- or walking-related lower-extremity symptoms, together with imaging evidence of central canal, lateral recess, or foraminal stenosis considered clinically relevant by the treating team. Supporting findings could include symptom aggravation with walking or lumbar extension, relief with flexion or rest, and corresponding MRI or CT evidence of stenotic narrowing.

3. Ankylosing spondylitis (AS)

Cases were classified as AS when the available records supported an inflammatory axial disease pattern and the final diagnosis had been established through rheumatologic or orthopedic evaluation. Operationally, this required documented chronic inflammatory back pain features and evidence of sacroiliac involvement on imaging and/or specialist-confirmed ankylosing spondylitis recorded in the patient chart. Where applicable, disease classification was aligned with concepts underlying the modified New York criteria, including radiographic sacroiliitis together with compatible clinical features such as inflammatory back pain or reduced spinal mobility.

4. Osteoporotic vertebral compression fracture (OVCF)

Cases were classified as OVCF when low back pain was associated with imaging evidence of vertebral compression fracture in an osteoporotic or fragility-fracture context. Supporting information included older age, known osteoporosis or osteopenia, low-energy trauma or no major trauma, focal vertebral tenderness, and radiographic, CT, or MRI findings consistent with osteoporotic vertebral collapse. Cases were adjudicated as OVCF only when the available records supported osteoporotic or fragility-related vertebral compression as the final diagnosis rather than malignant, infectious, or other secondary causes of collapse.

5. Infectious diseases of the spine (IDS)

Cases were classified as IDS when the records supported spinal infection, including pyogenic spondylitis, discitis, vertebral osteomyelitis, epidural abscess, or spinal tuberculosis, based on compatible clinical features together with imaging and/or laboratory evidence and subsequent specialist evaluation. Supporting findings could include persistent or progressive back pain, fever or constitutional symptoms, elevated inflammatory markers where available, and MRI or other imaging findings suggestive of spinal infection. MRI was treated as the most important imaging modality when available. Where applicable, microbiological results, pathological evidence, or treatment response were also considered, but not all cases required the same confirmatory test.

6. Metastatic spinal tumor (MST)

Cases were classified as MST when the available records supported metastatic involvement of the spine as the cause of the index presentation, based on clinical history, imaging findings, and subsequent oncologic or inpatient evaluation. Supporting evidence could include known malignancy or later-confirmed systemic malignancy, imaging findings suggestive of vertebral or epidural metastatic disease, and specialist assessment documenting spinal metastasis as the final diagnosis.

7. Multiple myeloma (MM)

Cases were classified as MM when the final diagnosis in the available records was consistent with accepted International Myeloma Working Group criteria and had been established through hematologic evaluation. Supporting evidence could include monoclonal protein studies, bone marrow assessment where available, imaging evidence of myeloma-related bone disease, and documented specialist-confirmed final diagnosis in the record. Because the study focused on initial low back pain presentation, these confirmatory data were used for reference adjudication but were not uniformly available to the models during evaluation.

8. Urinary system diseases (USD)

Cases were classified as USD when the final diagnosis indicated a non-musculoskeletal urinary system cause of the patient’s low back pain or flank/back-predominant symptoms, based on subsequent urologic evaluation, imaging, laboratory testing, and final clinical diagnosis. These cases were included to test whether the models could distinguish non-musculoskeletal conditions from MSDs and recommend an appropriate referral pathway. Cases were included only when the urinary system diagnosis had been clearly established in the documented course of care.

Not all disease categories in this study were confirmed by the same type of final test. Rather, adjudication was based on the most reliable diagnosis established from the complete available clinical record for each case. This reflects the retrospective outpatient-focused design of the study and was intended to provide a stable benchmark for comparative LLM evaluation, but it does not replace a uniform gold-standard verification process across all disease categories.
