# Supplementary material for: Performance of DeepSeek V3.2 and ChatGPT 5.1 in Musculoskeletal Triage and Differential Diagnosis of Outpatients With Low Back Pain: Multidimensional Comparative Study
Source: J Med Internet Res. 2026 Jul 3;28:e92315. doi: 10.2196/92315 (PMC13331072; doi:10.2196/92315)
Supplement: Multimedia Appendix 3 [file jmir-v28-e92315-s003.docx]

**Multimedia Appendix 4.** The main features and default inference parameters of the 2 state-of-the-art LLMs used in this study.

**DeepSeek V3.2**

DeepSeek V3.2 (released December 1, 2025) is a long-context sparse-attention LLM while delivering GPT-5-level reasoning and strong agent/tool-use capability [1]. Its distinguishing features include DeepSeek Sparse Attention (DSA) for efficient long-context processing, a scaled RL post-training pipeline (with a high-compute V3.2-Speciale variant reported to exceed GPT 5 and approach Gemini-3.0-Pro reasoning), and a large-scale agentic task synthesis pipeline that improves instruction compliance and generalization in interactive tool environments. It further emphasizes controlled output length with lower compute cost, and is reported to achieve gold-medal-level performance in the 2025 IMO/IOI [2].

**ChatGPT 5.1 (*OpenAI*)**

GPT 5.1 is OpenAI’s GPT-5-series iterative upgrade (announced November 12, 2025) that targets more reliable real-world execution across chat and developer workflows: in ChatGPT it introduces two selectable variants—GPT 5.1 Instant (optimized for faster, more fluid conversations) and GPT 5.1 Thinking (allocates more deliberate reasoning for harder queries)—while “Auto” routing can choose the best of these by default [3]. In the API, GPT 5.1 is positioned as a flagship model for coding and agentic tasks, offering a configurable reasoning.effort setting (eg, none/low/medium/high), a 400k context window and up to 128k output tokens, enabling long-document processing and multi-step tool/agent workflows; OpenAI’s developer release notes also emphasize adaptive reasoning, improved coding, and extended prompt caching up to 24 hours to reduce latency and cost in long-running, multi-turn session [4].

Reference:

[1] DeepSeek-V3.2: Efficient Reasoning & Agentic AI. Latest updated December 2025. Accessed December 1, 2025. [https://huggingface.co/deepseek-ai/DeepSeek-V3.2]

[2] DeepSeek-AI. DeepSeek-V3.2-Exp: Boosting Long-Context Efficiency with DeepSeek Sparse Attention [Internet]. GitHub repository; 2025 [cited 2025 Dec 30]. [https://github.com/deepseek-ai/DeepSeek-V3.2-Exp]

[3] OpenAI. GPT-5.1: A smarter, more conversational ChatGPT [Internet]. San Francisco (CA): OpenAI; 2025 Nov 12 [cited 2026 Jan 20]. Available from: https://openai.com/index/gpt-5-1/

[4] OpenAI. GPT-5.1 Model (OpenAI API documentation) [Internet]. San Francisco (CA): OpenAI; [cited 2026 Jan 20]. Available from: <https://platform.openai.com/docs/models/gpt-5.1>

Default inference parameters of DeepSeek V3.2 and ChatGPT 5.1 used in this study

| Parameters | DeepSeek V3.2 | ChatGPT 5.1 |
| --- | --- | --- |
| Temperature | 1 | 1 |
| Top_p | 0.95 | 1 |
| Max_tokens | 8192 | 1048 |
| Presence_penalty | 0 | 0 |
| Frequency_penalty | 0 | 0 |
| Top_k | 0 | NA |
| Min_p | 0 | NA |
| Repetition_penalty | 1 | NA |
