# Supplementary material for: Performance of DeepSeek V3.2 and ChatGPT 5.1 in Musculoskeletal Triage and Differential Diagnosis of Outpatients With Low Back Pain: Multidimensional Comparative Study
Source: J Med Internet Res. 2026 Jul 3;28:e92315. doi: 10.2196/92315 (PMC13331072; doi:10.2196/92315)

**Multimedia Appendix 5.** Structured Prompts of the large language models (LLMs) (DeepSeek V3.2 and ChatGPT 5.1) and a complete example

Phase I:

Assume you are an experienced specialist in musculoskeletal disorders and are familiar with the latest national and international diagnostic standards for low back pain. Based on the patient’s chief complaint “[insert chief complaint]”, first determine whether the condition is musculoskeletal in origin; answer only “Yes” or “No.” If the condition is not musculoskeletal, specify the most appropriate department to which the patient should be referred. If the condition is musculoskeletal, provide the single most likely diagnosis plus three differential diagnoses, and briefly justify why each differential requires consideration.

Phase II:

Assume you are an experienced specialist in musculoskeletal disorders and are familiar with the latest national and international diagnostic standards for low back pain. Based on the patient’s structured information “[insert structured information]”, first determine whether the condition is musculoskeletal in origin; answer only “Yes” or “No.” If the condition is not musculoskeletal, specify the most appropriate department to which the patient should be referred. If the condition is musculoskeletal, provide the single most likely diagnosis plus three differential diagnoses, and briefly justify why each differential requires consideration.

**a complete example:**

Phase I:


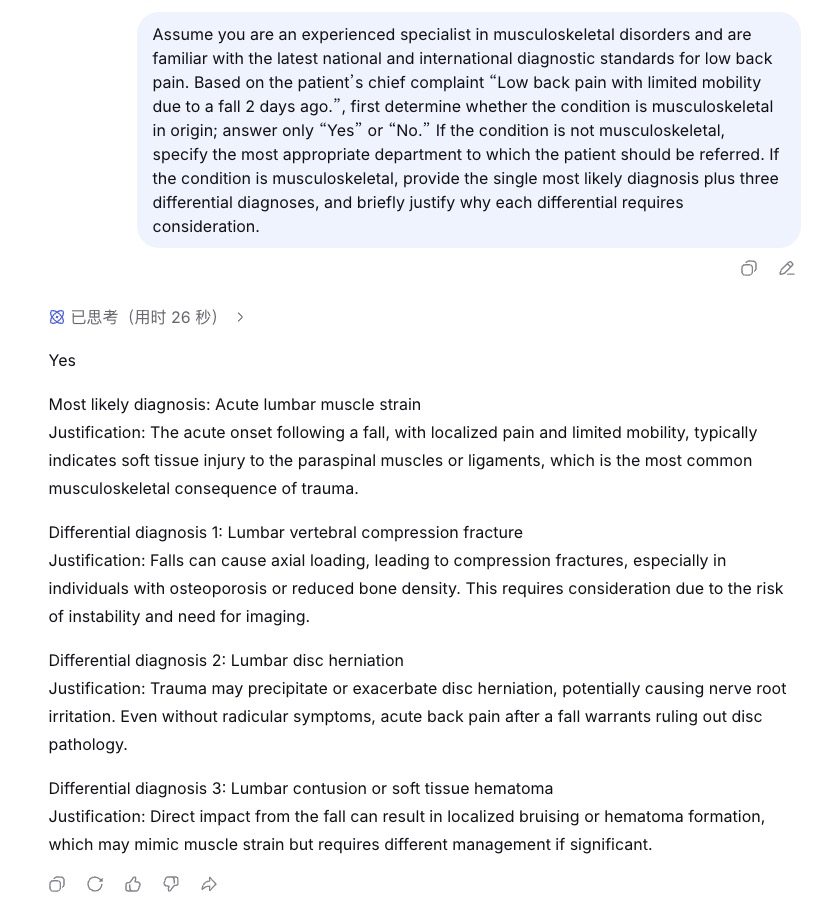


Phase II:


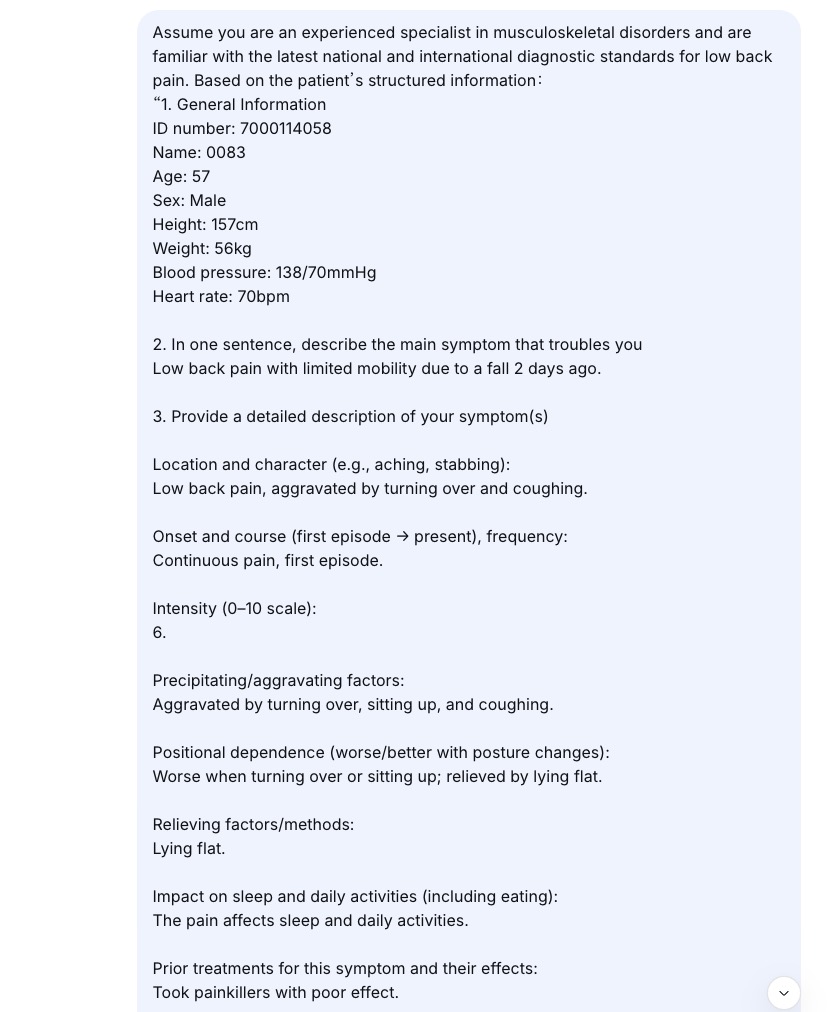

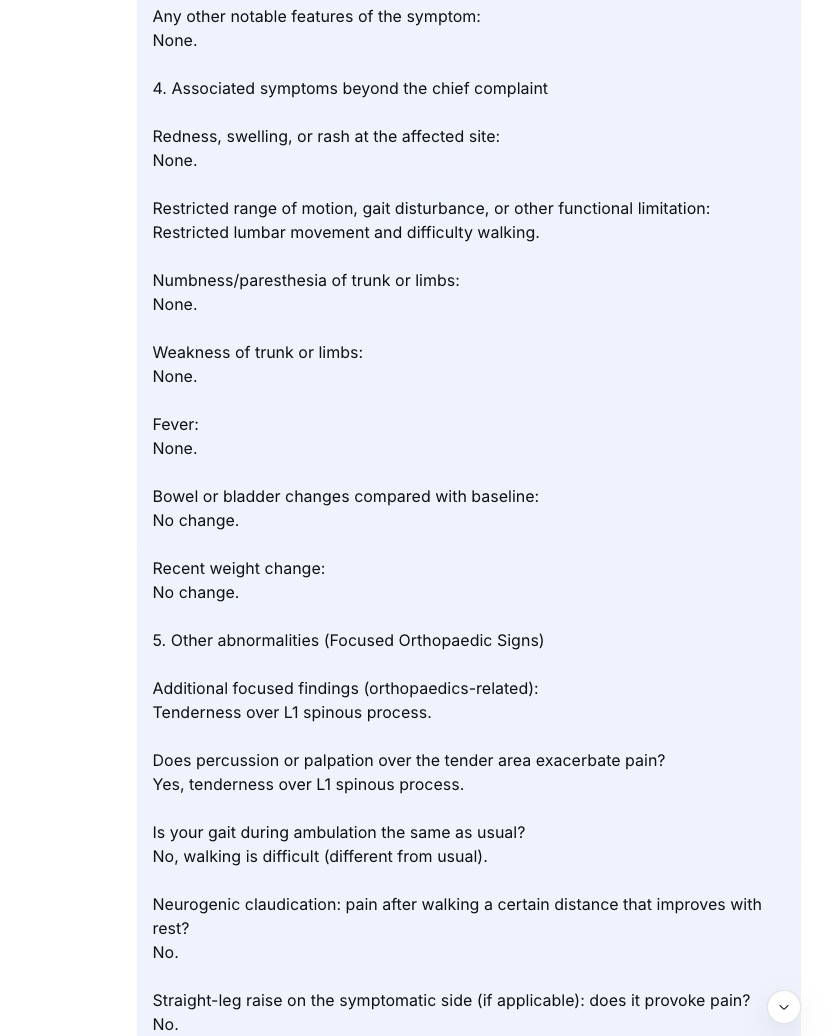

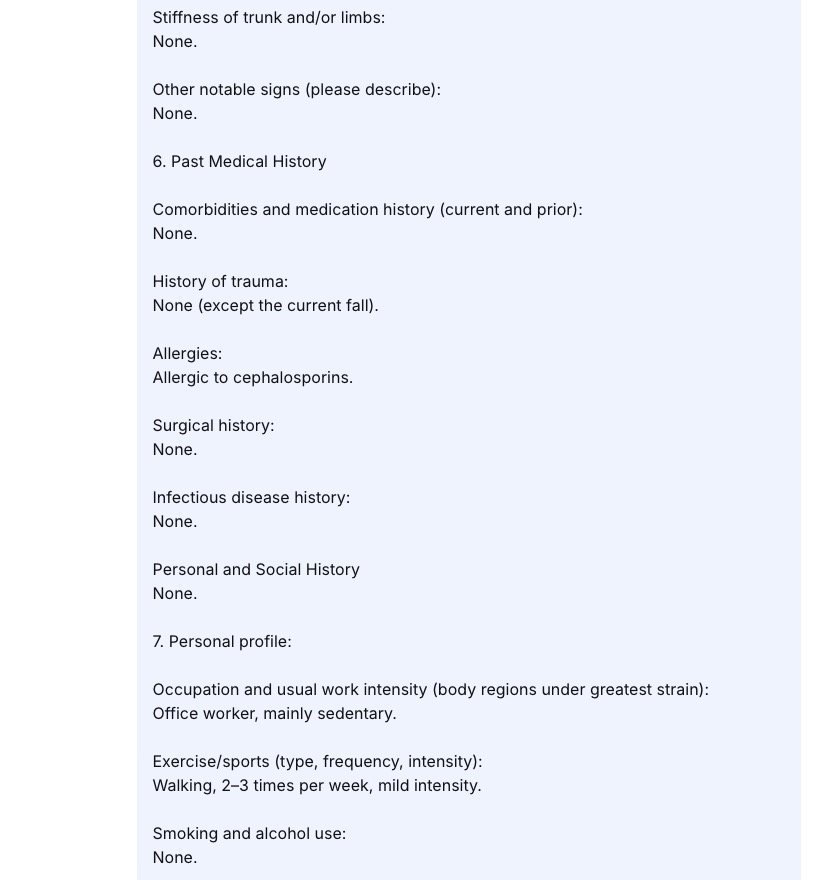

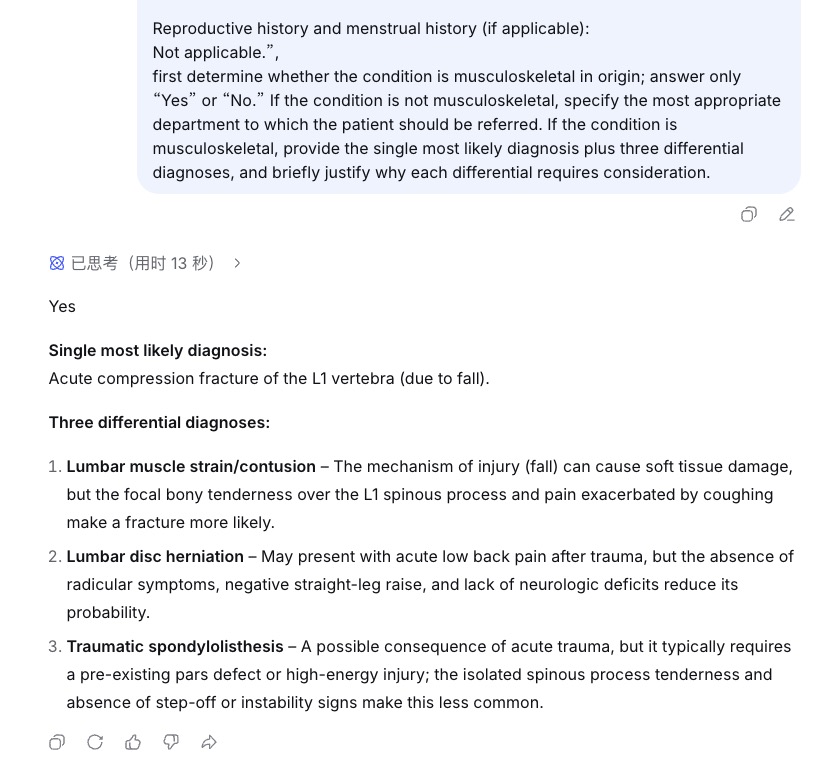

Supplement: Multimedia Appendix 4 [file jmir-v28-e92315-s004.docx]
