# Supplementary material for: Performance of DeepSeek V3.2 and ChatGPT 5.1 in Musculoskeletal Triage and Differential Diagnosis of Outpatients With Low Back Pain: Multidimensional Comparative Study
Source: J Med Internet Res. 2026 Jul 3;28:e92315. doi: 10.2196/92315 (PMC13331072; doi:10.2196/92315)
Supplement: Multimedia Appendix 5 [file jmir-v28-e92315-s005.docx]

**Patient History Structured Questionnaire**

1. **General Information**

- ID number:
- Name:
- Age:
- Sex:
- Height:
- Weight:
- Blood pressure:
- Heart rate:

1. **In one sentence, describe the main symptom that troubles you**

1. **Provide a detailed description of your symptom(s)**

- Location and character (e.g., aching, stabbing):
- Onset and course (first episode → present), frequency:
- Intensity (0–10 scale):
- Precipitating/aggravating factors:
- Positional dependence (worse/better with posture changes):
- Relieving factors/methods:
- Impact on sleep and daily activities (including eating):
- Prior treatments for this symptom and their effects:
- Any other notable features of the symptom:

1. **Associated symptoms beyond the chief complaint**

- Redness, swelling, or rash at the affected site:
- Restricted range of motion, gait disturbance, or other functional limitation:
- Numbness/paresthesia of trunk or limbs:
- Weakness of trunk or limbs:
- Fever:
- Bowel or bladder changes compared with baseline:
- Recent weight change:

1. **Other abnormalities (**Focused Orthopaedic Signs**)**

- Additional focused findings (orthopaedics-related):
- Does percussion or palpation over the tender area exacerbate pain?
- Is your gait during ambulation the same as usual?
- Neurogenic claudication: pain after walking a certain distance that improves with rest?
- Straight-leg raise on the symptomatic side (if applicable): does it provoke pain?
- Stiffness of trunk and/or limbs:
- Other notable signs (please describe):

1. **Past Medical History**

- Comorbidities and medication history (current and prior):
- History of trauma:
- Allergies:
- Surgical history:
- Infectious disease history:
- Personal and Social History

1. **Personal profile:**

- Occupation and usual work intensity (body regions under greatest strain):
- Exercise/sports (type, frequency, intensity):
- Smoking and alcohol use:
- Reproductive history and menstrual history (if applicable):
