# Supplementary material for: Performance of DeepSeek V3.2 and ChatGPT 5.1 in Musculoskeletal Triage and Differential Diagnosis of Outpatients With Low Back Pain: Multidimensional Comparative Study
Source: J Med Internet Res. 2026 Jul 3;28:e92315. doi: 10.2196/92315 (PMC13331072; doi:10.2196/92315)
Supplement: Multimedia Appendix 7 [file jmir-v28-e92315-s007.docx]

**Multimedia Appendix 8.** Demographic information of included patients.

| Disease | Lumbar spinal stenosis | Lumbar disc herniation | | Ankylosing spondylitis | Osteoporotic vertebral compression fracture | Infectious diseases of spine | Metastatic Spinal Tumor | Multiple myeloma | Urinary system diseases |
| --- | --- | --- | --- | --- | --- | --- | --- | --- | --- |
| Age | 60.81 ± 6.34 | | 52.65± 7.43 | 46.97 ± 5.18 | 66.22 ± 3.86 | 55.77 ± 7.08 | 63.98 ± 4.61 | 62.77 ± 5.22 | 54.18 ± 6.82 |
| Gender (M/F) | 10/10 | | 9/11 | 12/8 | 6/14 | 11/9 | 11/9 | 12/8 | 13/7 |
| BMI (kg/m^2^) | 21.83 ± 3.04 | | 21.65 ± 2.12 | 20.15 ± 1.91 | 24.95 ± 3.71 | 23.85 ± 2.26 | 21.45 ± 6.82 | 23.55 ± 2.02 | 24.01± 2.13 |
