# Supplementary material for: Performance of DeepSeek V3.2 and ChatGPT 5.1 in Musculoskeletal Triage and Differential Diagnosis of Outpatients With Low Back Pain: Multidimensional Comparative Study
Source: J Med Internet Res. 2026 Jul 3;28:e92315. doi: 10.2196/92315 (PMC13331072; doi:10.2196/92315)
Supplement: Multimedia Appendix 9 [file jmir-v28-e92315-s009.docx]

**Multimedia Appendix 10.** Comparison of the preliminary diagnosis accuracy of the large language models (LLMs) for low back pain. Preliminary diagnosis accuracy of DeepSeek V3.2 and ChatGPT 5.1 across six musculoskeletal etiologies of low back pain (n = 20 per etiology; total N = 120) under Phase I (chief complaint) and Phase II (structured questionnaire) inputs. In addition to accuracy (% correct), key paired comparisons are summarized using paired risk difference (RD) expressed in percentage points (%) and its 95% confidence interval (CI): (1) within-model Phase II vs Phase I, and (2) within-phase ChatGPT vs DeepSeek (paired by patient record). For each paired comparison, discordant pairs were defined as b (baseline correct → comparison incorrect) and c (baseline incorrect → comparison correct).

| Disease | DeepSeek V3.2 (Phase I) | ChatGPT 5.1 (Phase I) | DeepSeek V3.2 (Phase II) | ChatGPT 5.1 (Phase II) | RD (%) ^a^, 95% CI ^b^, *P* ^c^ (ChatGPT 5.1 [Phase I] vs DeepSeek V3.2 [Phase I]) | RD (%), 95% CI, *P*  (ChatGPT 5.1 [Phase II] vs DeepSeek V3.2 [Phase II]) | RD (%), 95% CI, *P* (DeepSeek V3.2, Phase II vs Phase I) | RD (%), 95% CI, *P* (ChatGPT 5.1, Phase II vs Phase I) |
| --- | --- | --- | --- | --- | --- | --- | --- | --- |
| Total | 48.3% | 35.0% | 76.7% | 87.5% | -13.3, [-22.5, -3.8], 0.011* | 10.8, [2.5, 19.2], 0.019* | 28.3, [16.8, 38.8], 0.001* | 52.5, [42.8, 60.6], <0.001* |
| Lumbar spinal stenosis | 40.0% | 25.0% | 75.0% | 80.0% | -15.0, [-34.2, 5.8], 0.375 | 5.0, [-6.9, 18.0], 1.000 | 35.0, [1.0, 60.2], 0.092 | 55.0, [29.1, 70.4], <0.001* |
| Lumbar disc herniation | 85.0% | 80.0% | 100.0% | 100.0% | -5.0, [-19.4, 7.8], 1.000 | 0.0, [-16.1, 16.1], 1.000 | 15.0, [-3.8, 36.0], 0.250 | 20.0, [-0.1, 41.6], 0.125 |
| Ankylosing spondylitis | 35.0% | 50.0% | 80.0% | 85.0% | 15.0, [-0.3, 28.8], 0.250 | 5.0, [-13.4, 23.8], 1.000 | 45.0, [20.6, 61.7], 0.004* | 35.0, [12.8, 53.3], 0.016* |
| Osteoporotic vertebral compression fracture | 60.0% | 10.0% | 85.0% | 100.0% | -50.0, [-67.6, -25.0], 0.002* | 15.0, [-3.8, 36.0], 0.250 | 25.0, [0.6, 46.3], 0.125 | 90.0, [64.2, 97.2], <0.001* |
| Infectious diseases of spine | 45.0% | 30.0% | 65.0% | 85.0% | -15.0, [-47.4, 21.7], 0.607 | 20.0, [-7.2, 43.9], 0.289 | 20.0, [-12.6, 47.7], 0.388 | 55.0, [29.1, 70.8], <0.001* |
| Metastatic Spinal Tumor | 25.0% | 15.0% | 55.0% | 75.0% | -10.0, [-26.2, 4.5], 0.500 | 20.0, [-13.0, 48.1], 0.388 | 30.0, [-2.7, 55.5], 0.146 | 60.0, [33.6, 74.9], <0.001* |

^a^ RD was computed as the paired risk difference in percentage points, calculated as (c−b)/N × 100%.

^b^ The 95% CI for paired RD was calculated using the Newcombe hybrid-score method for paired proportions based on the full 2 × 2 paired classification table.

^c^ Exact two-sided **McNemar p-values** were calculated from the discordant pairs using a binomial formulation, and exact p-values are reported where possible (*P* < 0.001 shown when applicable).

* Indicates a significant difference in McNemar’s test (*P* < 0.05).
