# Supplementary material for: Performance of DeepSeek V3.2 and ChatGPT 5.1 in Musculoskeletal Triage and Differential Diagnosis of Outpatients With Low Back Pain: Multidimensional Comparative Study
Source: J Med Internet Res. 2026 Jul 3;28:e92315. doi: 10.2196/92315 (PMC13331072; doi:10.2196/92315)
Supplement: Multimedia Appendix 10 [file jmir-v28-e92315-s010.docx]

**Multimedia Appendix 11.** Comparison of the differential diagnosis agreement of the large language models (LLMs) for low back pain. Differential diagnosis agreement of DeepSeek V3.2 and ChatGPT 5.1 across six musculoskeletal etiologies of low back pain (n = 20 per etiology; total N = 120) under Phase I (chief complaint) and Phase II (structured questionnaire) inputs. Because some cases did not proceed to differential diagnosis after incorrect triage, available sample sizes varied across comparisons and were not fully paired. In addition to mean ± SD, key comparisons are summarized using Hedges' g and its 95% confidence interval (CI): (1) within-phase ChatGPT 5.1 vs DeepSeek V3.2, and (2) within-model Phase II vs Phase I.

| Disease | DeepSeek V3.2 (Phase I), mean ± SD (n) ^a^ | ChatGPT 5.1 (Phase I), mean ± SD (n) | DeepSeek V3.2 (Phase II), mean ± SD (n) | ChatGPT 5.1 (Phase II), mean ± SD (n) | Hedges' g ^b^, 95% CI ^c^, *P* ^d^ (ChatGPT 5.1 [Phase I] vs DeepSeek V3.2 [Phase I]) | Hedges' g, 95% CI, *P* (ChatGPT 5.1 [Phase II] vs DeepSeek V3.2 [Phase II]) | Hedges' g, 95% CI, *P* (DeepSeek V3.2, Phase II vs Phase I) | Hedges' g, 95% CI, *P* (ChatGPT 5.1, Phase II vs Phase I) |
| --- | --- | --- | --- | --- | --- | --- | --- | --- |
| Total | 1.27 ± 0.71 (n=112) | 1.34 ± 0.70 (n=98) | 2.02 ± 0.74 (n=114) | 2.03 ± 0.77 (n=117) | 0.10, [-0.18, 0.37], 0.479 | 0.01, [-0.25, 0.27], 0.799 | 1.03, [0.76, 1.32], <0.001* | 0.93, [0.64, 1.24], <0.001* |
| Lumbar spinal stenosis | 0.89 ± 0.46 (n=18) | 1.27 ± 0.44 (n=15) | 1.90 ± 0.62 (n=20) | 2.40 ± 0.49 (n=20) | 0.79, [0.20, 1.38], 0.032* | 0.85, [0.27, 1.50], 0.014* | 1.75, [1.18, 2.68], <0.001* | 2.29, [1.66, 3.28], <0.001* |
| Lumbar disc herniation | 1.80 ± 0.60 (n=20) | 1.90 ± 0.62 (n=20) | 2.50 ± 0.74 (n=20) | 2.40 ± 0.58 (n=20) | 0.16, [-0.45, 0.79], 0.631 | -0.14, [-0.88, 0.43], 0.420 | 0.99, [0.35, 2.00], 0.003* | 0.79, [0.18, 1.54], 0.018* |
| Ankylosing spondylitis | 1.40 ± 0.66 (n=20) | 1.40 ± 0.73 (n=20) | 2.15 ± 0.65 (n=20) | 1.95 ± 0.67 (n=20) | 0.00, [-0.60, 0.63], 0.916 | -0.29, [-0.98, 0.33], 0.359 | 1.09, [0.52, 1.77], 0.002* | 0.75, [0.18, 1.36], 0.038* |
| Osteoporotic vertebral compression fracture | 1.42 ± 0.75 (n=19) | 1.14 ± 0.64 (n=14) | 2.20 ± 0.75 (n=20) | 2.10 ± 0.70 (n=20) | -0.37, [-1.11, 0.29], 0.291 | -0.13, [-0.82, 0.46], 0.560 | 0.99, [0.37, 1.88], 0.002* | 1.34, [0.74, 2.21], <0.001* |
| Infectious diseases of spine | 0.80 ± 0.54 (n=15) | 0.91 ± 0.79 (n=11) | 2.00 ± 0.47 (n=18) | 2.00 ± 0.63 (n=20) | 0.15, [-0.73, 1.01], 0.796 | 0.00, [-0.63, 0.63], 1.000 | 2.25, [1.52, 3.54], <0.001* | 1.48, [0.76, 2.63], 0.002* |
| Metastatic Spinal Tumor | 1.15 ± 0.65 (n=20) | 1.11 ± 0.46 (n=18) | 1.19 ± 0.39 (n=16) | 1.18 ± 0.86 (n=17) | -0.07, [-0.70, 0.56], 0.780 | -0.02, [-0.75, 0.66], 0.951 | 0.06, [-0.58, 0.68], 0.969 | 0.09, [-0.58, 0.79], 0.851 |

^a^ Differential diagnosis agreement was calculated only for cases in which the model first correctly classified the presentation as musculoskeletal and therefore proceeded to provide differential diagnoses. The n value reported in each model-phase column represents the number of correctly triaged musculoskeletal cases included in the corresponding mean ± SD calculation; for each Hedges’ g comparison, the denominators are the n values from the two model-phase columns being compared.

^b^ Hedges' g was calculated from the available cases for each comparison. Positive values indicate higher differential diagnosis scores in ChatGPT 5.1 than in DeepSeek V3.2 for within-phase comparisons, and in Phase II than in Phase I for within-model comparisons.

^c^ The 95% CI for Hedges' g was obtained by bootstrap resampling (4,000 resamples).

^d^ Two-sided p-values were calculated using the Mann-Whitney U test. Exact p-values are reported where possible (*P* < 0.001 shown when applicable).

* Indicates significant difference (*P* < 0.05).
