# Supplementary material for: Performance of DeepSeek V3.2 and ChatGPT 5.1 in Musculoskeletal Triage and Differential Diagnosis of Outpatients With Low Back Pain: Multidimensional Comparative Study
Source: J Med Internet Res. 2026 Jul 3;28:e92315. doi: 10.2196/92315 (PMC13331072; doi:10.2196/92315)
Supplement: Multimedia Appendix 11 [file jmir-v28-e92315-s011.docx]

**Multimedia Appendix 12**. Interrater agreements for performance evaluation of the large language models (LLMs).

| LLMs |  | Relevance | Understanding and reasoning | Groundedness | Trust and satisfaction | Harm |
| --- | --- | --- | --- | --- | --- | --- |
| DeepSeek V3.2 | ICC ^a^ | 0.773 | 0.852 | 0.781 | 0.943 | 0.875 |
|  | (95% CI) | (0.690-0.837) | (0.794-0.895) | (0.697-0.846) | (0.922-0.959) | (0.856-0.901) |
| ChatGPT 5.1 | ICC | 0.924 | 0.789 | 0.758 | 0.952 | 0.901 |
|  | (95% CI) | (0.896-0.945) | (0.672-0.862) | (0.670-0.825) | (0.935-0.965) | (0.897-0.912) |

^a^ Intraclass correlation coefficient (ICC)
