# Supplementary material for: Performance of DeepSeek V3.2 and ChatGPT 5.1 in Musculoskeletal Triage and Differential Diagnosis of Outpatients With Low Back Pain: Multidimensional Comparative Study
Source: J Med Internet Res. 2026 Jul 3;28:e92315. doi: 10.2196/92315 (PMC13331072; doi:10.2196/92315)
Supplement: Multimedia Appendix 12 [file jmir-v28-e92315-s012.docx]

**Multimedia Appendix 13.** Evaluation of the Large Language Models’ Rationales for Low Back Pain.

|  | DeepSeek V3.2 | ChatGPT 5.1 | *P* |
| --- | --- | --- | --- |
| Relevance | 4.08 ± 0.58 | 4.04 ± 0.79 | 0.641 |
| Understanding and reasoning | 4.14 ± 0.59 | 4.55 ± 0.56 | 0.011* |
| Groundedness | 4.88 ± 0.20 | 4.87 ± 0.30 | 0.133 |
| Trust and satisfaction | 3.96 ± 0.80 | 3.88 ± 0.82 | 0.572 |
| Harm | 4.18 ± 0.46 | 4.21 ± 0.44 | 0.454 |
