# Supplementary material for: Performance of DeepSeek V3.2 and ChatGPT 5.1 in Musculoskeletal Triage and Differential Diagnosis of Outpatients With Low Back Pain: Multidimensional Comparative Study
Source: J Med Internet Res. 2026 Jul 3;28:e92315. doi: 10.2196/92315 (PMC13331072; doi:10.2196/92315)

**Multimedia Appendix 14.** Number of safety-risk cases in 4 prespecified red-flag conditions by model and phase. Bars show the number of cases classified as safety-risk errors for osteoporotic vertebral compression fracture (OVCF), infectious diseases of the spine (IDS), metastatic spinal tumor (MST), and multiple myeloma (MM) in DeepSeek V3.2 and ChatGPT 5.1 under Phase I (chief complaint only) and Phase II (structured questionnaire) conditions.


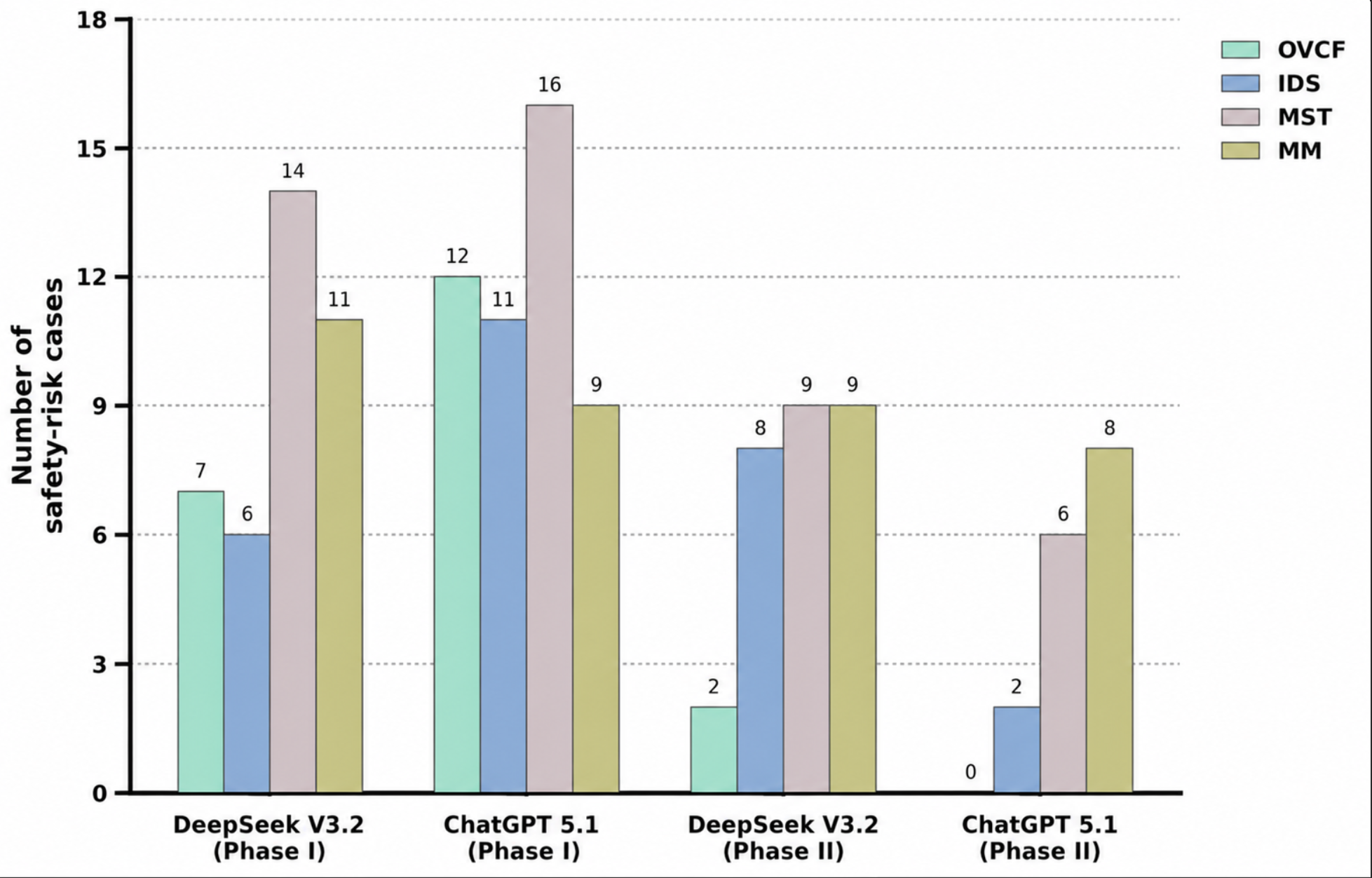

Supplement: Multimedia Appendix 13 [file jmir-v28-e92315-s013.docx]
